# Area-based breast percentage density estimation in mammograms using weight-adaptive multitask learning

**DOI:** 10.1038/s41598-022-16141-2

**Published:** 2022-07-14

**Authors:** Naga Raju Gudhe, Hamid Behravan, Mazen Sudah, Hidemi Okuma, Ritva Vanninen, Veli-Matti Kosma, Arto Mannermaa

**Affiliations:** 1 Institute of Clinical Medicine, Pathology and Forensic Medicine, Multidisciplinary Cancer Research community, University of Eastern Finland, P.O. Box 1627, 70211 Kuopio, Finland; 2 Department of Clinical Radiology, Kuopio University Hospital, P.O. Box 100, 70029 Kuopio, Finland; 3 Institute of Clinical Medicine, Radiology, Translational Cancer Research Area, University of Eastern Finland, P.O. Box 1627, 70211 Kuopio, Finland; 4 Biobank of Eastern Finland, Kuopio University Hospital, Kuopio, Finland

**Keywords:** Cancer, Computational biology and bioinformatics, Risk factors, Computer science

## Abstract

Breast density, which is a measure of the relative amount of fibroglandular tissue within the breast area, is one of the most important breast cancer risk factors. Accurate segmentation of fibroglandular tissues and breast area is crucial for computing the breast density. Semiautomatic and fully automatic computer-aided design tools have been developed to estimate the percentage of breast density in mammograms. However, the available approaches are usually limited to specific mammogram views and are inadequate for complete delineation of the pectoral muscle. These tools also perform poorly in cases of data variability and often require an experienced radiologist to adjust the segmentation threshold for fibroglandular tissue within the breast area. This study proposes a new deep learning architecture that automatically estimates the area-based breast percentage density from mammograms using a weight-adaptive multitask learning approach. The proposed approach simultaneously segments the breast and dense tissues and further estimates the breast percentage density. We evaluate the performance of the proposed model in both segmentation and density estimation on an independent evaluation set of 7500 craniocaudal and mediolateral oblique-view mammograms from Kuopio University Hospital, Finland. The proposed multitask segmentation approach outperforms and achieves average relative improvements of 2.88% and 9.78% in terms of F-score compared to the multitask U-net and a fully convolutional neural network, respectively. The estimated breast density values using our approach strongly correlate with radiologists’ assessments with a Pearson’s correlation of $r = 0.90$ (95% confidence interval [0.89, 0.91]). We conclude that our approach greatly improves the segmentation accuracy of the breast area and dense tissues; thus, it can play a vital role in accurately computing the breast density. Our density estimation model considerably reduces the time and effort needed to estimate density values from mammograms by radiologists and therefore, decreases inter- and intra-reader variability.

## Introduction

Breast cancer (BC) occurs with the highest incidence of all cancers in women across 27 European Union countries (1,237,588 new cancer cases with 28.7% being BC; 555,650 total cancer deaths with 16.5% due to BC in 2020) (https://ecis.jrc.ec.europa.eu/). Early detection of BC is a critical diagnostic requirement for lowering the BC mortality rate. Digital X-ray mammography is the gold standard and the most reliable imaging technique for BC screening in the early stages. The European Reference Organization for Quality Assured Breast Screening and Diagnostic Services recommends regular breast screenings for women over the age of 40 for both craniocaudal (CC)-view and mediolateral oblique (MLO)-view mammograms^1^.

Breast density has been identified as one of the strongest independent risk factors contributing to BC. Numerous studies demonstrated a positive association between breast density and BC risk^2–4^. The mammographic breast percentage density (PD) measures the relative amount of fibroglandular (also known as dense) tissue within the breast area. Women with higher PD values ($> 75\%$) have an indicative risk of BC that is 4-to-6 fold higher than women with lower PD values ($< 5\%$)^2^. The sensitivity of mammography is density dependent, i.e., the higher the density, the lower the sensitivity due to the masking effect^5^. The sensitivity is also affected by other factors, such as light intensity of the mammography machine, vendor specific processing protocol, perception errors, and the composition of the breast tissue^6^. In clinical practice, radiologists visually analyze the patterns and distribution of fibroglandular tissues within the mammograms and report the density scores following the Breast Imaging Reporting and Data System (BI-RADS)^7^. The 4th edition of BI-RADS categorizes the breast density into four quartiles in range of 0–100% with an increment of 25%. The assessment of breast density using BI-RADS 4th edition subjects to intra-reader variability^8^. To reduce this variability, the breast composition lexicon was updated in BI-RADS 5th edition. In the 5th edition, the qualitative analysis is replaced with subjectivity. With the highest incidence of BC, the need for the specialized radiologist is growing. Given shortage of expert radiologists and workload, substantial time and efforts are needed to examine mammograms at large scale. Moreover, the quantitative and qualitative assessments of the breast density by radiologists are subjective, leading to inter- and intra-reader variability^9^.

There is a growing interest among medical imaging experts in developing fully automated methods that can assess PD values in a robust and quantifiable fashion. Semiautomated approaches, such as Cumulus^10^ and DM-Scan^11^, have been developed for area-based PD estimation. However, these approaches require the domain expert (radiologist) to adjust a threshold value to segment the dense tissues for each mammogram, leading to the same problems as with manual assessments: time needed, subjective results, and intra- and inter-reader variability. Fully automatic software, such as LIBRA^12^ and Quantra^13^, have been developed for area-based and volumetric-based PD estimation, respectively. Despite being leading-edge tools, LIBRA and Quantra have a few limitations, such as over- or under-segmenting the fibroglandular tissues^14^. In many instances, LIBRA performs poorly in delineating the pectoral muscle from the breast region^14^, potentially due to data variability, different mammogram acquisition protocols, and vendor-specific post-processing techniques (in the case of processed mammograms). The pectoral muscle has similar pixel intensities and texture to the breast region, and the boundary separating the pectoral muscle and the breast region are usually obscure and irregular. Excluding the pectoral muscle from the breast region in MLO-view mammograms is another challenge that must be handled for accurate breast density estimation.

Traditional image processing techniques, such as thresholding^15^ and clustering^16^, have been well established for segmentation tasks. However, the major limitations of adopting traditional algorithms are selection of discriminative features in each given image for segmentation and finding an optimal threshold value to segment the fibroglandular tissues within the breast area. Artificial intelligence-based approaches, specifically deep learning (DL) algorithms based on convolutional neural networks (CNNs), have shown remarkable performances in various medical imaging applications^17–19^. The DL algorithms automatically extract the most descriptive and silent features within an image for a given task. The sophisticated computing infrastructure (graphical processing unit) enhances DL algorithms’ training and deployment in the clinical settings. Long et al.^20^ proposed an encoder–decoder-based fully convolutional neural networks (FCN) for the semantic segmentation task. The encoder captures the contextual and spatial information, and the decoder reconstructs the information and segments the regions of interest from the input mammogram. Inspired by this work^20^, Ronneberger et al.^19^ modified the FCN by introducing skip connections to concatenate features from the encoder to the corresponding decoder and named the architecture U-net. The gold-standard U-net architecture has been successfully incorporated in various biomedical image segmentation tasks. Despite its popularity, U-net has a few limitations, such as the loss of spatial information during concatenation of the features from the encoder to the decoder, and it often fails to segment regions of interest at different scales and variations^21,22^.

Recently, multitask learning (MTL) was employed to improve the performance of image segmentation tasks^23–25^. Parameter sharing between subtasks is the most common approach used to perform MTL, as it avoids recomputing each task’s parameters and thus improves computational speed by reducing memory usage^24^. MTL has been shown to have a higher generalization capability and to reduce overfitting^24,25^. Kendall et al.^23^ proposed an MTL framework with multiple regression and classification tasks for semantic segmentation and demonstrated that task-dependent homoscedastic uncertainty improves the representation and individual task performance.

A few studies have incorporated DL algorithms into the breast-density estimation task using digital mammograms^26–30^. Kallengberg et al.^30^ developed a sparse convolutional autoencoder to automatically extract features in a mammogram using an unsupervised learning technique. The learned features are fed to a simple neural network classifier for fatty- and dense-tissue classification^30^. They showed that the computed PD scores strongly correlate with the manual Cumulus scores ($r = 0.85$) and reported dice coefficients of $0.63 \pm 0.19$ and $0.95 \pm 0.05$ for the dense and fatty tissue segmentation. Lee et al.^14^ developed a fully automatic DL algorithm for PD estimation based on the FCN approach that segments the breast area and dense tissues in the mammograms. The study considered BI-RADS^7^ density ratings as ground truth and generated binary masks for the dense tissues. The PD values estimated by the algorithm showed Pearson correlation of $\textit{r} = 0.81$ and $r = 0.79$ for the CC- and MLO-view mammograms, respectively, compared with those estimated using LIBRA. Other studies^26–29^ used CNNs to classify the mammogram pixels into fatty and dense classes following BI-RADS 4th edition^31^ .

Previous studies segmented the breast area using classical edge-detection techniques or contour-based methods^32^. These approaches often failed to delineate the pectoral muscle and air gaps within the mammogram^33^. Mammograms acquired from various sources and sites often differ in terms of pixel intensities. Segmenting the dense tissues using image histogram thresholding based on BI-RADS categories increases the sensitivity of the model^14^. Accurate segmentation of breast and dense tissues are vital to computing the PD values. The main contributions of this study are listed as follows:

1. We proposed a multitask DL architecture, named MTLSegNet, that simultaneously segments the breast area and the dense tissues within a given mammogram and further computes the PD values using the segmented regions.
2. We generated 31,731 (21,315 from Kuopio University Hosptial (KUH) dataset and 10,416 from open-sourced datasets) breast-area and dense-tissue ground-truth binary masks for the segmentation task, under the supervision of two expert radiologists from KUH. The KUH data with annotations are available by request.
3. We evaluated the segmentation performance of the proposed approach against multitask U-net and FCN, as baseline approaches. We also compare the estimated PD values with existing LIBRA and Quantra software.

## Material and methods

### Data acquisition protocol

The study was approved by the ethics committee of KUH. For the purposes of this retrospective image analysis, the need for patient consent was waived by the Chair of the Hospital District. All experiments were conducted according to the relevant guidelines and the principles expressed in the Declaration of Helsinki.

The KUH mammograms were acquired using Selenia Dimensions from Hologic, Inc. or AMULET Innovality from FUJIFILM corporation. The mammograms from the Kuopio region (Finland) were retrieved from the picture archiving and communications system. Pseudonymized digital mammograms (for Presentation) in DICOM format were collected from 6278 women from January 2011 to December 2020. Two expert radiologists with experience of 25 and 15 years reviewed all the mammograms, and any with a visual appearance of implants, marker clips, or device names, which considerably damage the breast region, were excluded. The resulting KUH dataset contains mammograms from 5682 women (21,315 mammograms).

**Figure 1:**
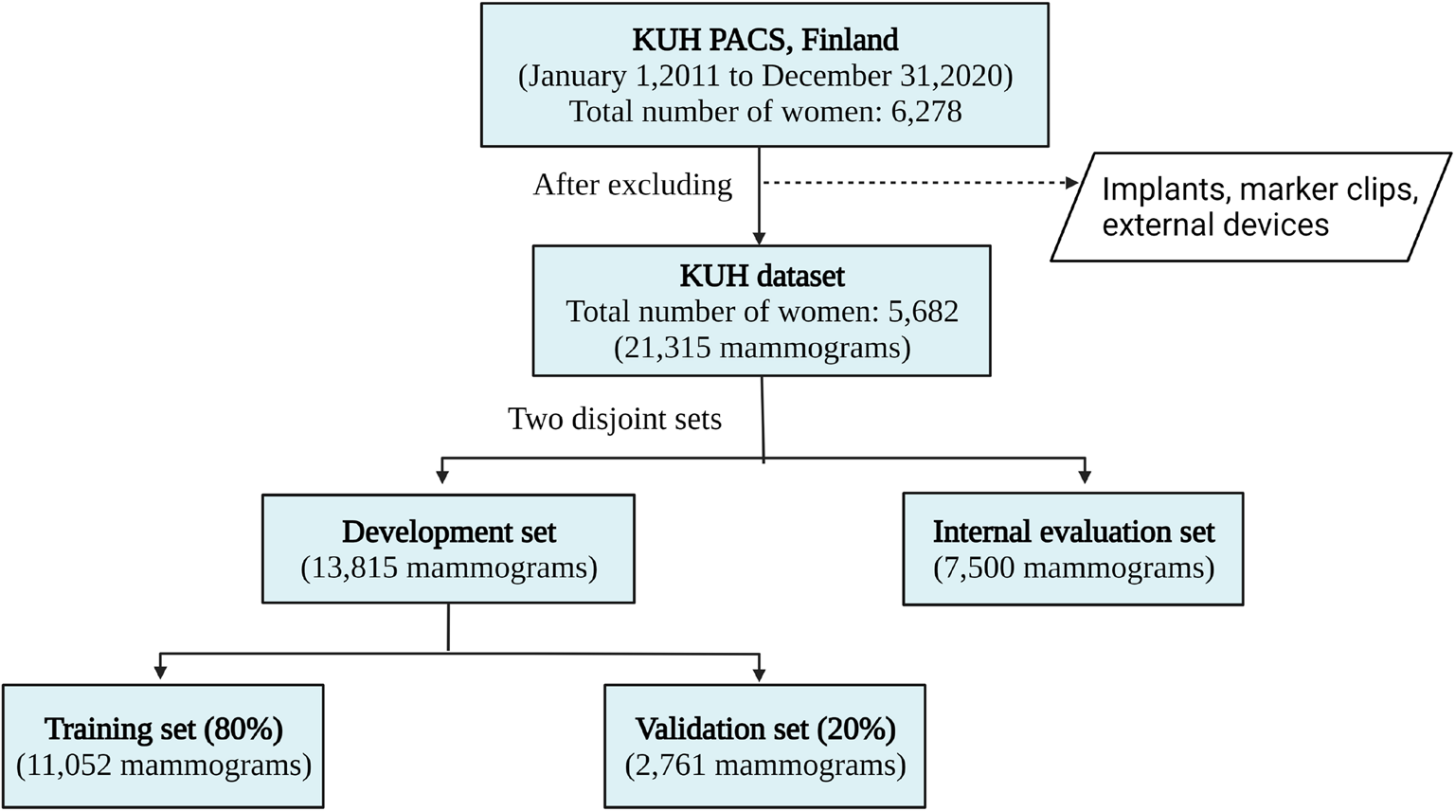
Flowchart describing the KUH dataset preparation. After excluding mammograms containing implants, marker clips, and/or external devices, we divided the KUH dataset into two disjoint sets: the development set and the internal evaluation set.

Figure 1 shows the flowchart for preparing the KUH mammogram dataset. The KUH dataset was randomly divided into two disjoint sets, one for model development and the second for evaluating the model performance. The development set contained 13,815 mammograms and was further divided into a training set (80%; 11,052 mammograms) and validation set (20%; 2763 mammograms). The evaluation set contained 7500 mammograms and was used to demonstrate the performance of our proposed and baseline segmentation models, and to estimate the breast PD values. Note that the internal evaluation set is not used for the model development and training. To avoid data leakage, we ensured that mammograms from the same patients only appear in one set during the KUH data split.

To address the data variability problem, we also included three publicly available datasets: the Mammographic Image Analysis Society digital mammogram dataset (MIAS)^34^, mini-DDSM^35^, and INbreast^36^. The MIAS and mini-DDSM are open-source datasets, and for the INbreast dataset, we obtained written agreement from the authors for use in research. MIAS is the most requested dataset in the mammography research community. The original dataset was digitized with a 50-micron pixel edge. It consists of 322 digitized MLO-view mammograms from 161 women at $1024\times 1024$ pixels, each with corresponding label information. The mini-DDSM is the lightweight version of the Digital Database for Screening Mammography (DDSM)^37^, containing 9684 mammograms from 2421 women (mean age of 57.51 ± 12.71) with variable image dimensions between 125 and 320 pixels. INbreast is a full-field digital mammography dataset consisting of 410 mammograms from 115 women. The KUH data splitting protocol for the three publicly available datasets resulted in few test mammograms for model evaluation, especially in the MIAS and INbreast datasets. Therefore, we followed a 60:20:20 splitting protocol for training, validation, and evaluation for the MIAS, mini-DDSM, and INbreast datasets to maintain data distribution harmony and have a handful of mammograms to evaluate the performance of the proposed and the baseline approaches. Table 1 summarizes the datasets and the data splitting protocol used in this study. In total, we used 30% (9582) of the mammograms from the KUH and publicly available datasets for evaluation.

**Table 1:** The splitting protocol used in this study. We employed a 60:20:20 splitting protocol for training, validation, and evaluation sets for the MIAS, INbreast, and mini-DDSM datasets.

Dataset	Development set				Evaluation set	
	Training set ($n = 17{,}304$)		Validation set ($n = 4845$)		Test set ($n = 9582$)	
	CC	MLO	CC	MLO	CC	MLO
KUH ($n = 21{,}315$)	5526	5526	1382	1381	3750	3750
MIAS ( $n = 322$)	N. A	194	N. A	64	N. A	64
mini-DDSM ($n = 9684$)	2906	2906	968	968	968	968
INbreast ($n = 410$)	123	123	41	41	41	41

Note that the splitting protocol for the KUH dataset is different than the public datasets. *N.A* not available.

**Figure 2:**
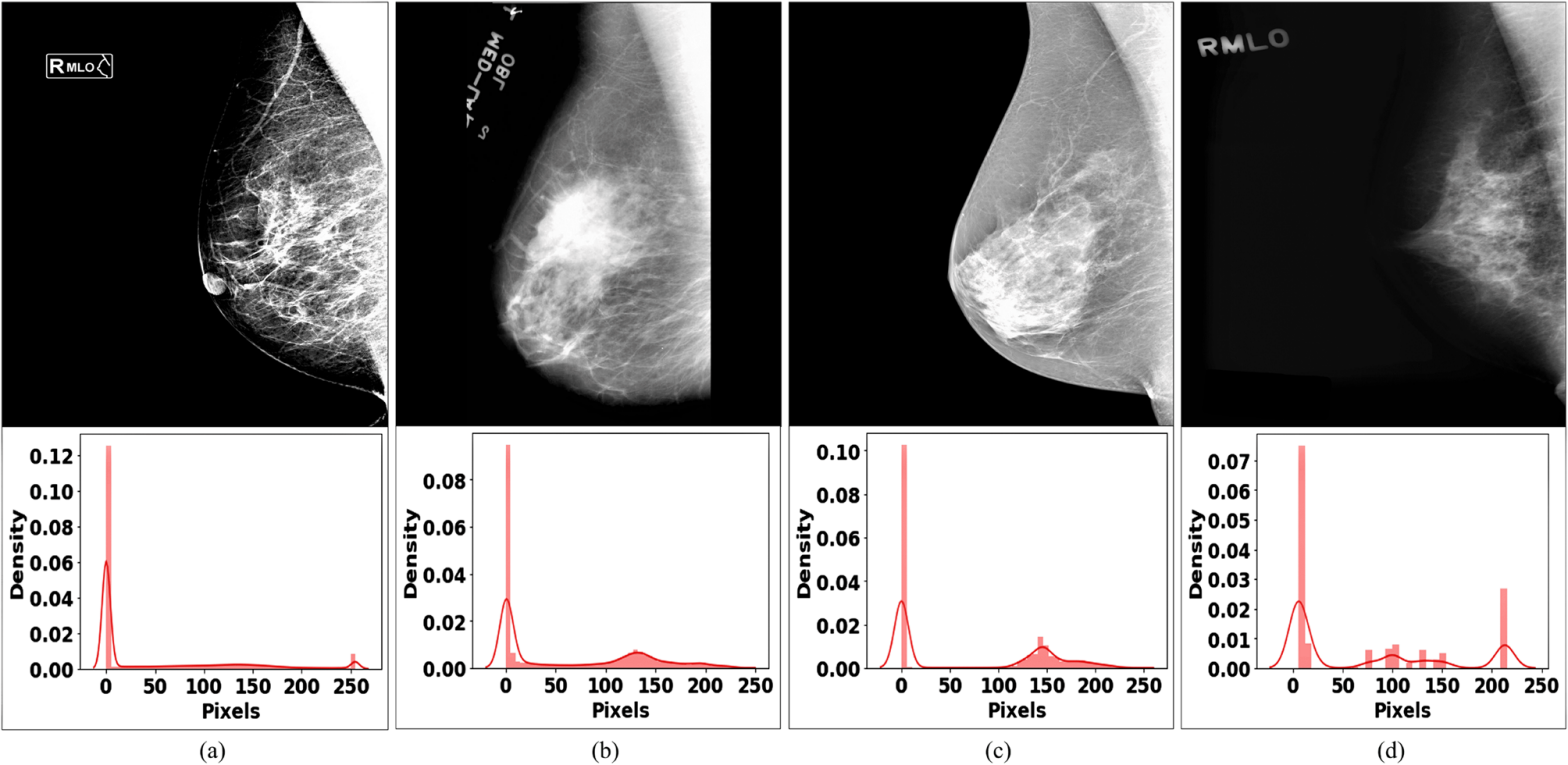
Examples of mammograms from the datasets used in this study. (**a**) KUH, (**b**) MIAS, (**c**) INbreast, and (**d**) mini-DDSM. The histogram of the grayscale value distribution for each mammogram is given below the corresponding mammogram.

The datasets used in this study vary in the number of mammograms and the image pixel intensity distribution, as illustrated in Fig. 2. The variation could be potentially due to the vendor-specific processing of the mammograms and the acquisition protocols. We combined the datasets and normalized the mammograms using the normalizer technique^38^ to develop a robust model capable of handling data variability issues. Normalization is a standard pre-processing technique that changes the range of pixel intensities of the individual image pixels and achieves consistency for the combined datasets.

### Ground truth annotations and reference PD value computation

Under the guidance of two expert radiologists from KUH, we developed an in-house mammogram annotation tool and generated in total 31,731 breast-area and dense-tissue binary masks, among which 21,315 are from the KUH dataset and 10,416 from the publicly available datasets, used in this study. All the annotations were reviewed by an experienced BC radiologist from KUH.

#### Breast-area segmentation mask

The contour-based algorithms remove the background noise (labels, patient identification, visible markers) and segment the breast region^39^. Although contour-based approaches are simple and easy to use, breast-region segmentation is challenging, especially for the MLO-view mammograms, due to the similar intensity of the pectoral muscle and the breast region. Furthermore, the visual partition line between the pectoral tissue and the breast area is obscure and often irregular in shape. We manually segmented the breast area using the VGG Image Annotator software tool^40^ and generated the annotations into JSON format, using OpenCV python package (https://docs.opencv.org/4.x/index.html), we generated the binary breast-area masks. We used the generated breast-area mask and overlapped it with the original image to remove the background noise. All the background pixels were set to zero, and the intensity range of the breast area was normalized using the min-max normalization technique.

#### Dense-tissue segmentation mask

**Figure 3:**
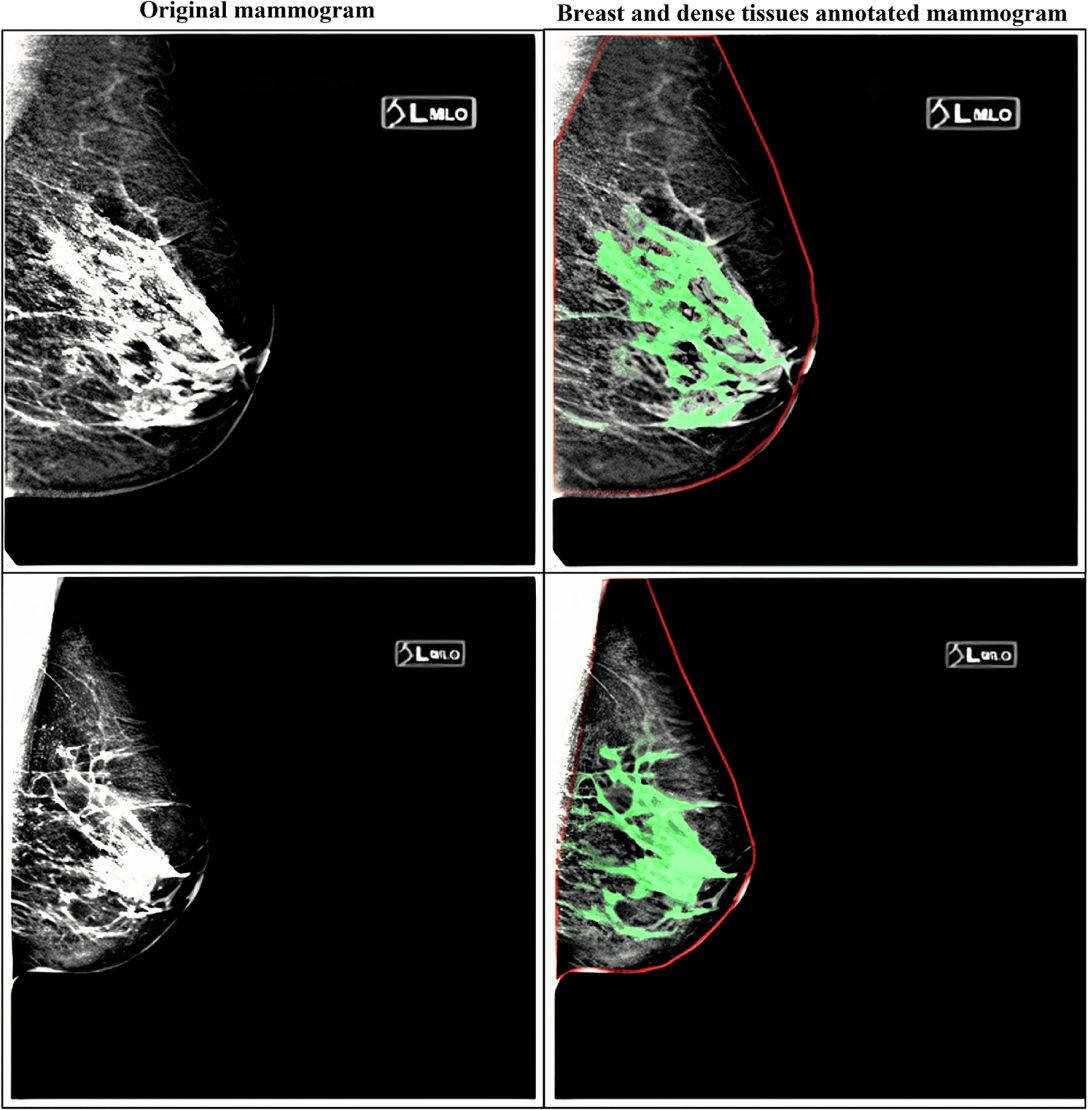
Examples of generated ground-truth segmentation masks from the KUH development set for the breast and dense-tissue segmentation. We overlapped the segmented binary masks on the original mammogram.

Based on BI-RADS categories, dense tissues are segmented using image-thresholding techniques^14^. With the variability in the image intensities and uncertainty in BI-RADS classification, the dense tissues are either over- or under-segmented. This study generated the dense-tissue binary masks using an in-house web-based interactive image segmentation tool developed in Python 3.6 and the Flask (https://flask.palletsprojects.com/en/2.0.x/) web framework under expert radiologist supervision. Figure 3 shows ground-truth annotations generated for the KUH dataset. The red contour line separates the breast area from other muscles, such as pectoral tissue in the MLO-view mammograms. The green pixels represent the dense (fibroglandular) tissues in the mammograms.

#### Reference PD value computation

For the KUH evaluation set (7,500 mammograms), two expert radiologists assessed the PD values, with an inter-reader correlation coefficient of 0.89. We have provided the Bland-Altman agreement plot between the two radiologists in Appendix A. We considered a difference of $\pm \,5\%$ PD value between the radiologists’ given PD values, a clinically acceptable difference (CDI); 6840 out of 7,500 KUH evaluation mammograms are within the CDI. The estimated PD values by the two radiologists within the CDI range were then averaged and used as a reference PD value for a given mammogram. For LIBRA and Quantra, we computed the PD values using LIBRA software tool version 1.0.4 and Selenia Dimensions^®^, Hologic Inc., Bedford machine equipped with Quantra tool, respectively.

### Proposed architecture

**Figure 4:**
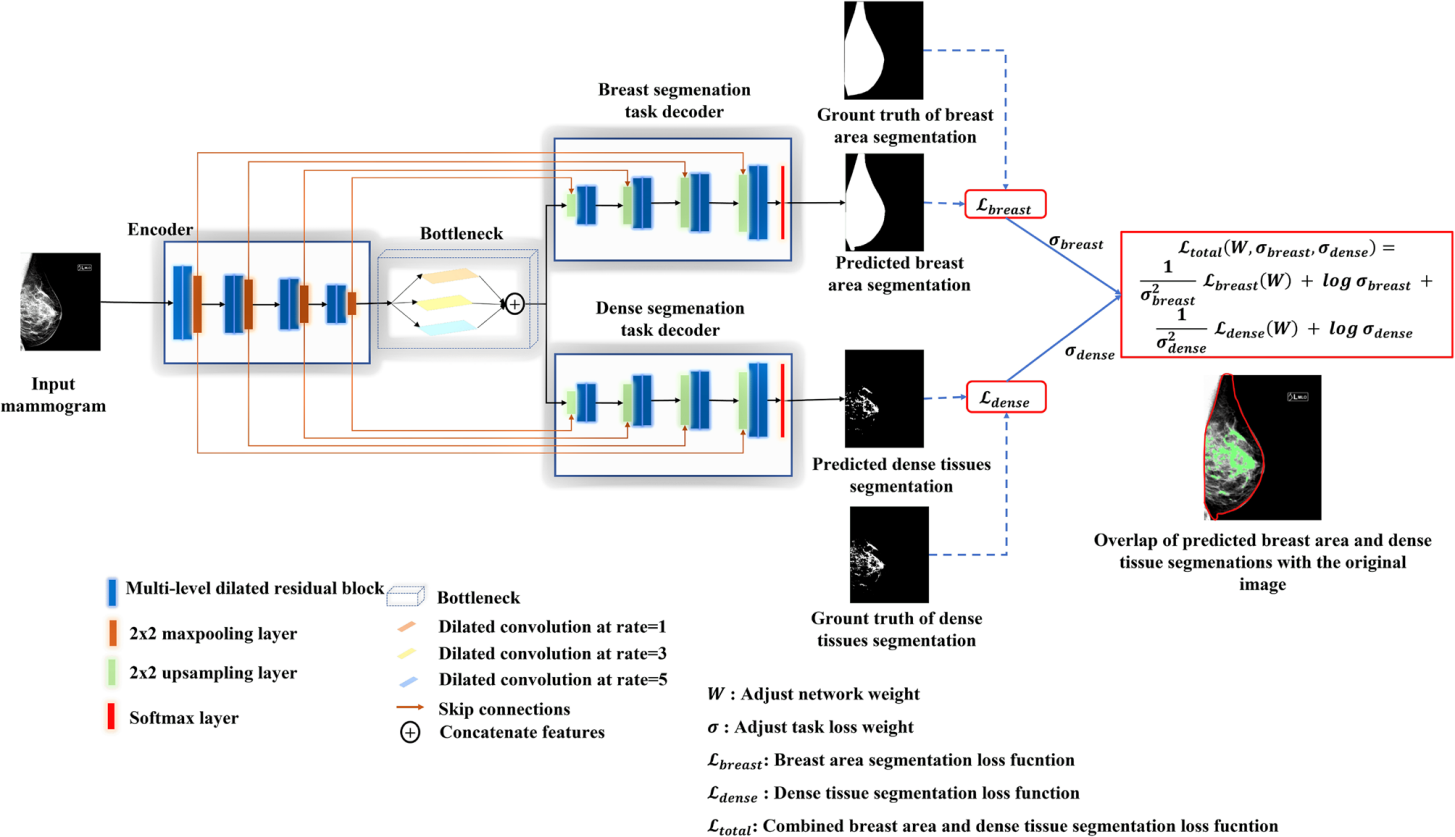
Illustration of the MTLSegNet architecture with encoder, bottleneck, and task-specific decoders. The encoder unit extracts the low- and high-level imaging features from the input mammogram. The extracted features are then fed into the bottleneck unit. The bottleneck unit further enhances the field of view by extracting more complex and spatial information at different resolutions. The task-specific decoders segment the breast area and the dense tissues, simultaneously. The loss function (focal Tversky loss^43^) is computed using the corresponding predicted and ground-truth segmentations of the breast area and the dense tissues. We modified the weight adaptive multi-task loss function^23^ for the segmentation task and computed the combined loss of the breast-area and dense-tissue segmentation tasks. The predicted segmentation outputs are overlapped with the original mammogram. The red contour line is the predicted segmented breast area, and the solid green pixels represent the predicted fibroglandular tissues within the breast area.

An overview of the proposed MTLSegNet architecture is illustrated in Fig. 4. MTLSegNet is based on the MTL approach with two task-specific networks to segment the breast area and the dense tissue simultaneously. We considered the dense-tissue segmentation as the main task and the breast-area segmentation as the auxiliary task. This helps the model better differentiate the breast area from other tissues, such as pectoral and abdominal tissue, in the MLO-view mammograms and enhances the dense-tissue segmentation within the breast area. The task-specific decoders share parameters with the encoder network, and the depth of the encoder network is similar to that of the U-net encoder network. We replaced the conventional blocks in the encoder and decoder paths with multilevel dilated residual blocks, as suggested in Gudhe et al.^22^, to enhance the learning capabilities of the network.

Additionally, we introduced three parallel dilated convolutions^41^ with dilation rates of *d* = 1, 3, and 5 as a bottleneck that expands the field of view by extracting more complex and spatial information at different resolutions. The decoder part of each task has up-sampling layers with transpose convolutions. The extracted features from the encoder are concatenated with the corresponding decoder layer, and the nonlinear residual skip connections restore the information loss during the transition of up-sampling features to down-sampling in the decoder^22^. The prediction layer of the individual tasks is a $1\times 1$ convolution layer activated by SoftMax^42^ as a nonlinear function that predicts the probability maps of the breast area and the dense tissues. We modified the weighted multitask loss function^23^ to compute the combined loss of the breast-area and the dense-tissue segmentation tasks.

#### Weight-adaptive multitask learning

Multitask learning is an inductive-transfer learning approach that improves generalization by sharing domain information among multiple tasks^23–25^. MTLSegNet consists of a common encoder and two decoders for the breast-area and dense-tissue segmentation. The features extracted from the encoder are shared by the two independent tasks.

Consider a dataset $\mathrm {D}= \{(x^{(i)},y_b^{(i)},y_d^{(i)})\}_{i=1}^{M}$, where $x^{(i) }$ represents the input mammogram of the instance *i*, and $y_b^{(i) }$ and $y_d^{(i) }$ are the breast-area and the dense-tissue ground-truth binary masks of the corresponding instance *i*. The learning function $\mathrm {F}$ of the MTL approach is represented as $\mathrm {F}(x^{(i)}, \theta _b^{(i)}, \theta _d^{(i)} )$, where $\theta _b^{(i)}$ and $\theta _d^{(i)}$ are the network’s weight parameters for the independent breast-area and dense-tissue segmentation tasks, respectively. The total energy function ${{{\textbf {E}}}}_{\mathrm {total}}$ is defined as follows:

1 $$\begin{aligned} {{{\textbf {E}}}}_{\mathrm {total}} = \lambda _{b} {{{\textbf {E}}}}_b (\theta _b )+ \lambda _d {{{\textbf {E}}}}_d (\theta _d ) \end{aligned}$$

where ${{{\textbf {E}}}}_b$ and ${{{\textbf {E}}}}_d$ are the energy functions, and $\lambda _b$ and $\lambda _d$ are non-negative hyperparameters with arbitrary values between (0,1]. Generally, the values of $\lambda _b$ and $\lambda _d$ are manually chosen until the model generalization is optimized, the so-called naïve approach. The parameter selection for the naïve approach is difficult and involves considerable computation, while the trained model usually becomes biased toward a specific task^23^.

The naïve multitask loss function is defined as follows^23^:

2 $$\begin{aligned} {{{\textbf {L}}}}_{\mathrm {total}} (\mathrm {D}: \theta _b, \theta _d) = \lambda _b \times {{{\textbf {L}}}}_b (\mathrm {D}: \theta _b) + \lambda _d \times {{{\textbf {L}}}}_d (\mathrm {D}: \theta _d). \end{aligned}$$

Since the $\lambda _b$ and $\lambda _d$ values are in the range (0, 1] and $\lambda _b + \lambda _d \le 1$, for simplicity, Equation (2) can be rewritten as:

3 $$\begin{aligned} {{{\textbf {L}}}}_{\mathrm {total}} (\mathrm {D}: \theta _b, \theta _d) = \lambda \times {{{\textbf {L}}}}_b (\mathrm {D}: \theta _b) + (1- \lambda ) \times {{{\textbf {L}}}}_d (\mathrm {D}: \theta _d). \end{aligned}$$

Motivated by^23,44^ , we modified the weight uncertainty loss function for segmenting the breast area and the dense tissues. The relative task weights, $\lambda _b$ and $\lambda _d$, are learned by considering the uncertainty in the output predictions of each individual task. We define the weight-adaptive multitask loss function ${{{\textbf {L}}}}_{total}$ as follows:

4 $$\begin{aligned} {{{\textbf {L}}}}_{\mathrm {total}} (\mathrm {D}: \theta , \sigma _b, \sigma _d) = {{{\textbf {L}}}}_b(\mathrm {D}: \theta , \sigma _b) + {{{\textbf {L}}}}_d(\mathrm {D}: \theta , \sigma _d) \end{aligned}$$

where ${{{\textbf {L}}}}_b$ and ${{{\textbf {L}}}}_d$ are the loss functions for the breast-area and dense-tissue segmentations, with $\sigma _b$ and $\sigma _d$ as the corresponding task weights for $\lambda _b$ and $\lambda _d$, respectively. Consider the likelihood of the model for each segmentation task as a scaled version of the model output $\mathrm {f}^\theta (x)$ with uncertainty $\sigma$, and $\theta$ as a network weight parameter squashed through a SoftMax function:

5 $$\begin{aligned} p (y|\mathrm {f}^\theta (x), \sigma ) = \mathrm {softmax}\left( \frac{1}{\sigma ^{2}} \mathrm {f}^\theta (x)\right) . \end{aligned}$$

Using the negative log-likelihood, the segmentation loss with uncertainty is expressed as follows:

6 $$\begin{aligned} \mathrm {log} \; p (y= c| \mathrm {f}^\theta (x), \sigma ) = \frac{1}{\sigma ^{2}} \mathrm {f}^\theta (x) - \mathrm {log} \; \Sigma _{c^{'}} \mathrm {exp}\left( \frac{1}{\sigma ^{2}} \mathrm {f}_{c^{'}}^\theta (x)\right) \end{aligned}$$

where $\mathrm {f}_{c^{'}}^\theta (x)$ is the *c*th element of the vector $\mathrm {f}^\theta (x)$ .

The multitask loss function ${{{\textbf {L}}}}_{\mathrm {total}} (\theta , \sigma _b, \sigma _d)$ is defined as:

7 $$\begin{aligned} \begin{aligned} {{{\textbf {L}}}}_{\mathrm {total}}(\theta , \sigma _b, \sigma _d)&= - \mathrm {log} \; p(y_b, y_d = c |~\mathrm {f}^\theta (x))\\&= \mathrm {log} \; p(y_b = c ; \mathrm {f}^\theta (x), \sigma _b) + \mathrm {log} \; p(y_d = c ; \mathrm {f}^\theta (x), \sigma _d)\\&= \frac{1}{\sigma _b^{2}} - \mathrm {log} \; \Sigma _{c^{'}} \mathrm {exp}\left( \frac{1}{\sigma _b^{2}} \mathrm {f}_{c^{'}}^\theta (x)\right) + \frac{1}{\sigma _d^{2}} - \mathrm {log} \; \Sigma _{c^{'}} \mathrm {exp}\left( \frac{1}{\sigma _d^{2}} \mathrm {f}_{c^{'}}^\theta (x)\right) \\&\approx \frac{1}{\sigma _b^{2}} {{{\textbf {L}}}}_b(\theta ) + \mathrm {log} \sigma _b + \frac{1}{\sigma _d^{2}} {{{\textbf {L}}}}_d(\theta ) + \mathrm {log} \sigma _d. \end{aligned} \end{aligned}$$

We employed the focal Tversky loss function (FTL)^43^ for each individual task, ${{{\textbf {L}}}}_b$ and ${{{\textbf {L}}}}_d$.

For each pixel *j*, $y^{(j)}_{b}$ and $y^{(j)}_{d}$ are the ground truths for the breast area and dense tissue, respectively, and ${\hat{y}}^{(j)}_{b}$ and ${\hat{y}}^{(j)}_{d}$ are the corresponding predicted segmentation masks. The FTLs for the breast-area and dense-tissue segmentation tasks are then defined as follows:

8 $$\begin{aligned} {{{\textbf {L}}}}_b= & {} \Sigma _c \left( 1- \left( \frac{\Sigma _{j=1}^{N} {\hat{y}}^{(j)_c}_{b} \; y^{(j)_c}_{b} + \varphi }{\Sigma _{j=1}^{N} {\hat{y}}^{(j)_c}_{b} \; y^{(j)_c}_{b} + \alpha \; \Sigma _{j=1}^{N} {\hat{y}}^{(j)_{{\bar{c}}}}_{b} \; y^{(j)_c}_{b}\; + \beta \; \Sigma _{j=1}^{N} {\hat{y}}^{(j)_c}_{b} \; y^{(j)_{{\bar{c}}}}_{b} + \varphi }\right) \right) ^{\frac{1}{\gamma }} \end{aligned}$$

9 $$\begin{aligned} {{{\textbf {L}}}}_d= & {} \Sigma _c \left( 1- \left( \frac{\Sigma _{j=1}^{N} {\hat{y}}^{(j)_c}_{d} \; y^{(j)_c}_{d} + \varphi }{\Sigma _{j=1}^{N} {\hat{y}}^{(j)_c}_{d} \; y^{(j)_c}_{d} + \alpha \; \Sigma _{j=1}^{N} {\hat{y}}^{(j)_{{\bar{c}}}}_{d} \; y^{(j)_c}_{d}\; + \beta \; \Sigma _{j=1}^{N} {\hat{y}}^{(j)_c}_{d} \; y^{(j)_{{\bar{c}}}}_{d} + \varphi }\right) \right) ^{\frac{1}{\gamma }} \end{aligned}$$

where *c* and ${\bar{c}}$ denote two class labels for region of interest and background, respectively. The total number of pixels in an image is denoted by *N*and $\varphi$ prevents division by zero. The hyperparameters $\alpha$ and $\beta$ can be tuned to improve the recall in case of class imbalance. The hyperparameter $\gamma$ represents the focal parameter for detecting hard classes with lower probabilities. We used $\alpha = 0.3$, $\beta = 0.7$, and $\gamma = 1$ as penalties, as suggested in^43^.

#### Computing area-based percentage mammogram density

The outputs of MTLSegNet are the probability scores of the breast-area and dense-tissue segmentations. We applied a threshold of 0.5 to convert the probability scores into binary masks. We resized the output predictions to their original image dimensions, as the reconstructed spatial resolutions are less than the original image spatial resolution due to the down-sampling and up-sampling operations. The PD value is computed as follows:

10 $$\begin{aligned} PD = \frac{\Sigma _{j=1}^{N} \; {\hat{y}}^{(j)}_{d}}{\Sigma _{j=1}^{N} \; {\hat{y}}^{(j)}_{b}} \times 100 \end{aligned}$$

where $\hat{y_b}$ and $\hat{y_d}$ are the predicted breast-area and dense-tissue segmentation binary masks containing the white pixels only.

## Implementation, evaluation metrics, and statistical analysis

### Implementation details

We implemented the proposed and the baseline approaches, multitask U-net and FCN, in Python 3.6 using PyTorch 1.3.1^45^, as the DL framework. Additionally, we implemented Otsu thresholding^46^, as a conventional approach, to segment fibroglandular tissues. We considered four steps to estimate PD value of a given mammogram using Otsu thresholding: first, we generated the segmented breast area following the protocol described in "Ground truth annotations and reference PD value computation" *Breast-area segmentation mask* and converted the segmented breast area into a grayscale image. In second step, we smoothened the grayscale segmented breast area using a Gaussian blur with a kernel size of 5. Then, we applied Otsu thresholding to segment the fibroglandular tissues. Finally, using Eq. (10), we computed the PD value.

The datasets considered in this study were acquired from different devices, introducing variability to the image dimensions, intensity, and visual appearance. We resized all the images to $256\times 256$ dimensions using bicubic interpolation to maintain the original aspect ratio. We combined all the validation set of mammograms from all datasets and fine-tuned the proposed model to find the optimal hyperparameters, including optimizer, learning rate, learning rate schedulers, and loss functions. We implemented the Bayesian optimization technique^47^ using an adaptive experimentation platform^48^ to find the optimal hyperparameters for the proposed model, the experiment results are provided in Appendix B. Additionally, we investigated the impact of various normalization techniques, including batch normalization^49^, instance normalization^50^, group normalization^51^, and weight standard normalization^52^, at batch sizes of 2, 4, 8, and 16 on the performance of the multitask segmentation models using the validation set of all the datasets. The normalization techniques accelerate the training process of DL models and help them converge faster^49^. The batch size and normalization experiment results are presented in Appendix C.

The proposed MTLSegNet and the baseline approaches were trained using the optimal hyperparameters for 100 epochs on a machine equipped with an Nvidia Tesla V100 16GB graphic card on an Intel Xeon processor provided by the IT Service Centre for Science (CSC) Finland^53^. The implementation source codes are available at https://gitlab.com/rajgudhe.uef/mtlsegnet.

### Segmentation evaluation metrics

We evaluated the segmentation performance of MTLSegNet and the baseline models using F-score and intersection over union (IoU). For a given image *x*, let *y* and ${\hat{y}}$ be the ground-truth and predicted binary masks, respectively. The evaluation metrics are defined as follows:

11 $$\begin{aligned} \mathrm {Precision}= & {} \frac{\Sigma _{j=1}^{N} \; {\hat{y}}^{(j)} \cap y^{(j)}}{\Sigma _{j=1}^{N} {\hat{y}}^{(j)}} \end{aligned}$$

12 $$\begin{aligned} \mathrm {Recall}= & {} \frac{\Sigma _{j=1}^{N} \; {\hat{y}}^{(j)} \cap y^{(j)}}{\Sigma _{j=1}^{N} y^{(j)}} \end{aligned}$$

13 $$\begin{aligned} \mathrm {F-score}= & {} \frac{2 \times \mathrm {Precision} \times \mathrm {Recall}}{\mathrm {Precision} + \mathrm {Recall}} \end{aligned}$$

14 $$\begin{aligned} \mathrm {IoU}= & {} \frac{\Sigma _{j=1}^{N} \; {\hat{y}}^{(j)} \cap y^{(j)}}{\Sigma _{j=1}^{N} \; {\hat{y}}^{(j)} \cup y^{(j)}} \end{aligned}$$

### Statistical evaluation of the estimated breast-density values

To determine the degree of association between the estimated PD values of MTLSegNet and baseline models with the radiologists provided reference PD values, Pearson’s correlation coefficients^54^ *r* at 95% confidence intervals (CI) were computed for each mammogram view. Bland–Altman plots^55^ was used to measure the limits of agreement (LoA) between the density estimation models at 95% CI.

## Results

In this section, we demonstrate the quantitative and qualitative performance of MTLSegNet for both segmentation and PD value estimation. For the breast-area and dense-tissue segmentations, we compare the performance of MTLSegNet against the baseline approaches, FCN and U-net. Furthermore, we compare the accuracy of the MTLSegNet-estimated PD values with the radiologist-provided, LIBRA-computed, and Quantra-computed PD values. Model evaluation is given for both CC- and MLO-views and the combined CC-MLO view mammograms. The CC-MLO view is formed by randomly shuffling the CC- and MLO-view mammograms from the evaluation sets of all datasets. We then combined the CC- and MLO-view mammograms from the same patients and created the CC-MLO-view evaluation set.

### Performance of breast and dense-tissue segmentation using MTLSegNet and baseline approaches

#### Weight-adaptive multitask learning outperforms the naïve multitask learning approach

**Figure 5:**
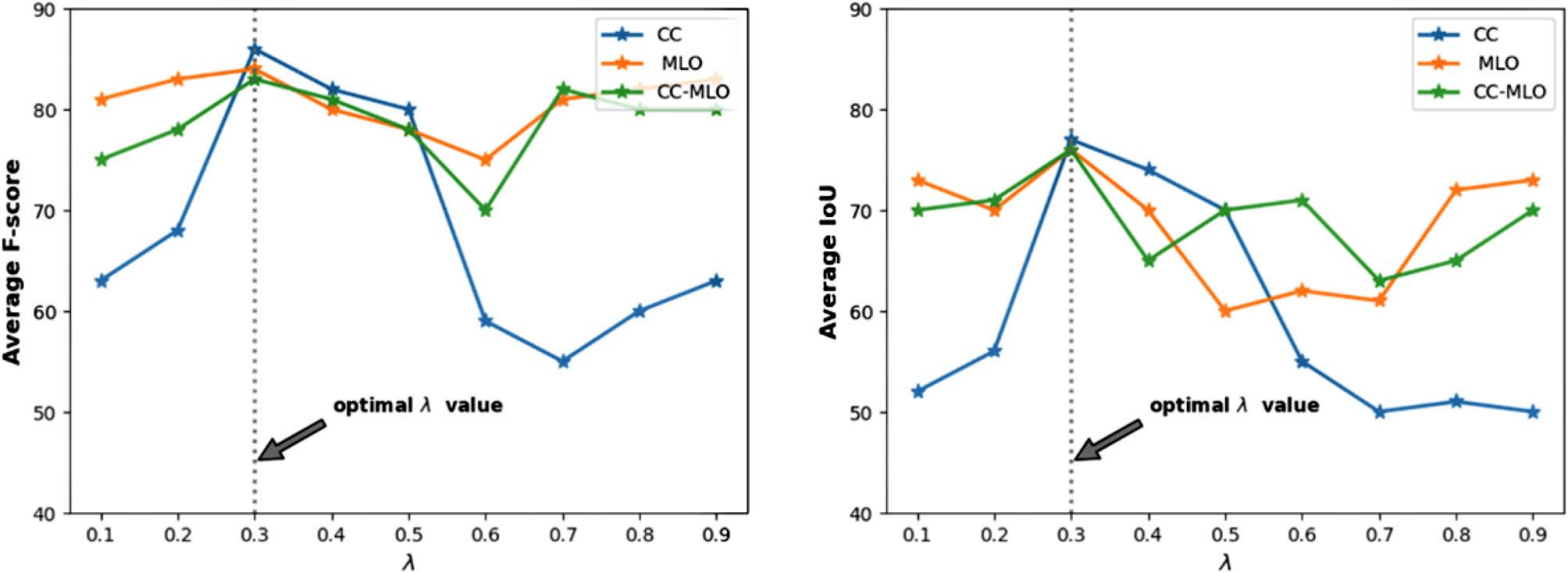
The performance of the naïve multitask loss function on the combined validation set. The weight parameter $\lambda$ in the range 0.1–0.9 is on the x-axis, and the segmentation evaluation metrics, IoU and F-score, are on the y-axis. The segmentation model trained with $\lambda = 0.3$ shows better performance compared to the other $\lambda$ values.

In this section, we demonstrate the efficacy of the weight-adaptive MTL, Eq. (7), compared to the naïve MTL, Eq. (3). For the naïve MTL, we implemented the trial-and-error approach, with $\lambda$ values in the range (0,1). Figure 5 shows the segmentation accuracy of different values of $\lambda$ in terms of F-score and IoU on the combined validation sets. The model trained with $\lambda = 0.3$ shows a better average segmentation performance for the CC-, MLO-, and CC-MLO-view mammograms.

The naïve multitask approach is an expensive grid search and time-consuming approach to find the optimal value of $\lambda$. We compared the performance of the modified weight-adaptive multitask loss function with the optimal weight parameter $\lambda = 0.3$ of the naïve multitask loss function. Table 2 shows that the modified weight-adaptive loss function performs better in segmenting the breast area and the dense tissues than the naïve approach for the CC-MLO-view of the combined datasets, with relative improvements of 10.15% and 14.23% in terms of F-score and IoU, respectively. The advantage of using the weight-adaptive multitask loss function is that the model automatically estimates the weight parameters by considering the weighted uncertainty parameter $\sigma$ of both the breast-area and the dense-tissue segmentations, which is considerably computationally less inexpensive than the naïve approach. It also reduces the bias between the primary task (dense-tissue segmentation) and the auxiliary task (breast-area segmentation).

**Table 2:** The comparison of naïve multitask loss function with the modified weight-adaptive multitask loss function. The model trained with weight-adaptive multitask loss function shows superior performance in segmenting the breast area and the dense tissues than the naïve approach, with relative improvements of 10.15% and 14.23% in terms of F-score and IoU, respectively, on the CC-MLO-view mammograms of the combined validation sets.

Combined validation set ($n = 4845$)	Naïve multitask		Weight-adaptive multitask	
	F-score	IoU	F-score	IoU
CC ($n = 2391$)	85.2	76.9	**85.9**	**78.3**
MLO ($n = 2454$)	83.5	75.2	**91.1**	**84.2**
CC-MLO ($n = 4845$)	83.7	75.2	**92.2**	**85.9**

Best values are in [bold].

#### Multitask learning shows superior performance compared to single-task segmentation

We trained MTLSegNet and the baseline methods for both single-task and multitask segmentations. For the single-task segmentation, we removed the second decoder from the architectures and trained all models with the optimal hyperparameters (more details are enclosed in Appendix B) for 100 epochs. We compared the performance of the individual tasks, the breast-area and the dense-tissue segmentations, against the multitask learning approach using the combined validation sets. For the multitask learning, we computed the evaluation metrics independently for the two task-specific decoders.

**Table 3:** The multitask learning approach shows superior segmentation performance compared to the single-task approach. For the multitask learning approach, we computed the evaluation metrics independently on the predictions of the two task-specific decoders. We compared the dense-tissue segmentation of CC-MLO-view mammograms for both the single-task and multitask approaches of FCN, U-net, and MTLSegNet (highlighted in bolditalics).

Combined validation set (*n* = 4845)			Single task		Multitask	
Model	Tissue	view	F-score	IoU	F-score	IoU
FCN	Breast	**CC **(*n* = **2391)**	83.15 ± 0.013	73.16 ± 0.001	80.31 ± 0.037	70.19 ± 0.003
		**MLO**(*n* = **2454**)	82.68 ± 0.001	70.38 ± 0.003	76.51 ± 0.003	72.72 ± 0.001
		**CC-MLO **(*n* = **4845**)	80.16 ± 0.019	63.16 ± 0.003	79.36 ± 0.003	70.32 ± 0.003
	***Dense***	**CC **(*n* = **2391**)	68.39 ± 0.02	58.16 ± 0.015	70.16 ± 0.016	68.26 ± 0.12
		**MLO **(*n* = **2454**)	65.62 ± 0.001	57.82 ± 0.013	73.19 ± 0.032	67.37 ± 0.220
		***CC-MLO*** (***n*** = ***4845***)	***65.13 ± 0.*002**	***57.10 ± 0.0012***	***73.03 ± 0***.**16**	***67***.**03 ± *0***.***11***
U-net	Breast	**CC **(*n* = **2391**)	87.32 ± 0.011	87.13 ± 0.003	84.19 ± 0.002	74.16 $\pm$ 0.009
		**MLO **(*n* = **2454**)	86.31 ± 0.003	86.09 ± 0.003	84.73 ± 0.005	72.78 ± 0.0042
		**CC-MLO**(*n* = ** 4845)**	87.51 ± 0.003	83.32 ± 0.001	85.13 ± 0.005	71.56 ± 0.019
	***Dense***	**CC **(*n* = ** 2391**)	71.53 ± 0.002	62.15 ± 0.013	73.36 ± 0.113	72.61 ± 0.001
		**MLO **(*n* = ** 2454**)	70.31 ± 0.002	61.92 ± 0.03	71.58 ± 0.056	70.32 ± 0.063
		***CC-MLO*** (*n* = ***4845)***	***68***.***19 ± 0***.***003***	***56***.***12 ± 0***.***017***	***70***.***19*** ± ***0***.***005***	***68.13 ± 0***.***007***
MTLSegNet	Breast	**CC **(*n* = ** 2391)**	89.181 ± 0.003	88.381 ± 0.006	85.840 ± 0.001	82.286 ± 0.001
		**MLO **(*n* = **2454)**	89.098 ± 0.004	88.223 ± 0.009	88.906 ± 0.008	87.852 ± 0.016
		**CC-MLO** (*n* = **4845**)	89.095 ± 0.003	88.212 ± 0.007	88.940 ± 0.007	87.915 ± 0.013
	***Dense***	**CC **(*n* = ** 2391)**	73.651 ± 0.03	68.899 ± 0.038	81.866 ± 0.013	75.041 ± 0.021
		**MLO **(*n* = **2454)**	70.195 ± 0.037	64.690 ± 0.035	81.965 ± 0.014	75.202 ± 0.022
		***CC-MLO*** (*n* = ***4845)***	***72.278 ± 0.022***	***67.140 ± 0.023***	***82.042 ± 0.010***	***75.323 ± 0.016***

Best values are in [bold].

Table 3 shows that the proposed multitask MTLSegNet approach outperforms the single-task approach in segmenting dense tissues by average relative improvement of 13.5% in terms of F-score on the CC-MLO-view mammograms. For the single-task approach, MTLSegNet outperforms the FCN and U-net dense-tissue segmentations by relative improvements of 10.97% and 5.99% in terms of F-score, respectively. Regarding the multitask approach, MTLSegNet shows superior performance for the dense-tissue segmentation compared to the multitask FCN and U-net by relative improvements of 12.34% and 16.88% in terms of F-score, respectively. We also notice that the breast-area segmentation by the single-task model performs slightly better than the multitask approach. Note that in the multitask segmentation approach, the model simultaneously segments the breast area and the dense tissues. To reduce the bias between the two tasks, the weight parameter of the individual decoder networks is balanced by the weight-adaptive loss function. In the single-task approach, the loss function is generalized to a specific task (e.g., the breast-area segmentation), thus achieving slightly better accuracy.

#### MTLSegNet outperforms the multitask segmentation U-net and FCN models

Table 4 shows that the proposed MTLSegNet approach outperforms the Otsu, FCN and U-net networks in all datasets. Average relative improvements of 24.07%, 3.17% and 2.29% in terms of F-score are observed over the Otsu, FCN and U-net, respectively, in the combined CC-MLO-view evaluation set of all datasets. The highest segmentation improvements with MTLSegNet over the DL approaches are attributed to the CC-MLO-view images of the KUH evaluation data, at 9.78% and 2.88% relative improvements over the FCN and U-net networks, respectively. Similarly, the lowest segmentation improvements with MTLSegNet are attributed to the CC-view of the mini-DDSM dataset, at 1.08% and 0.11% relative improvements over the FCN and U-net networks, respectively.

**Table 4:** The proposed MTLSegNet segmentation approach outperforms the FCN and U-net networks in all datasets. Numbers in parentheses denote the number of evaluation data points in each dataset. We compare the performance of MTLSegNet on the individual validation datasets for CC-, MLO-, and CC-MLO-view mammograms in terms of F-score and IoU, as evaluation metrics.

Evaluation set ($n = 9582$)	View	Model	F-score	IoU
KUH ($n = 7500$)	CC	Otsu	$55.32 \pm 0.31$	$47.51 \pm 0.03$
		FCN	$71.56 \pm 0.11$	$60.21 \pm 0.13$
		U-net	$80.09 \pm 0.07$	$70.48 \pm 0.08$
		MTLSegNet	${{\textbf {81.27}}} \pm {{\textbf {0.06}}}$	${{\textbf {71.95}}} \pm {{\textbf {0.07}}}$
	MLO	Otsu	$62.56 \pm 0.03$	$51.36 \pm 0.05$
		FCN	$75.91 \pm 0.05$	$65.41 \pm 0.06$
		U-net	$79.65 \pm 0.05$	$69.59 \pm 0.06$
		MTLSegNet	${{\textbf {80.41}}} \pm {{\textbf {0.04}}}$	${{\textbf {70.83}}} \pm {{\textbf {0.05}}}$
	CC-MLO	Otsu	$68.16 \pm 0.02$	$57.39 \pm 0.07$
		FCN	$75.32 \pm 0.06$	$65.76 \pm 0.21$
		U-net	$80.37 \pm 0.13$	$70.53 \pm 0.22$
		MTLSegNet	${{\textbf {82.69}}} \pm {{\textbf {0.02}}}$	${{\textbf {73.40}}} \pm {{\textbf {0.02}}}$
MIAS ($n = 64$)	MLO	Otsu	$44.37 \pm 0.02$	$51.56 \pm 0.11$
		FCN	$75.51 \pm 0.14$	$65.10 \pm 0.14$
		U-net	$78.28 \pm 0.10$	$67.77 \pm 0.10$
		MTLSegNet	${{\textbf {78.75}}} \pm {{\textbf {0.13}}}$	${{\textbf {68.41}}} \pm {{\textbf {0.13}}}$
mini-DDSM ($n = 1936$)	CC	Otsu	69.72± 0.03	76.32± 0.01
		FCN	$92.09 \pm 0.00$	$85.82 \pm 0.01$
		U-net	$92.98 \pm 0.01$	$87.27 \pm 0.02$
		MTLSegNet	${{\textbf {93.09}}} \pm {{\textbf {0.01}}}$	${{\textbf {87.43}}} \pm {{\textbf {0.02}}}$
	MLO	Otsu	$31.52 \pm 0.06$	$57.56 \pm 0.02$
		FCN	$75.51 \pm 0.14$	$65.10 \pm 0.14$
		U-net	$78.28 \pm 0.10$	$67.77 \pm 0.10$
		MTLSegNet	${{\textbf {78.75}}} \pm {{\textbf {0.13}}}$	${{\textbf {68.41}}} \pm {{\textbf {0.13}}}$
	CC-MLO	Otsu	$75.32 \pm 0.04$	$71.56 \pm 0.05$
		FCN	$91.78 \pm 0.00$	$85.23 \pm 0.01$
		U-net	$92.70 \pm 0.01$	$86.71 \pm 0.02$
		MTLSegNet	${{\textbf {92.83}}} \pm {{\textbf {0.01}}}$	${{\textbf {86.92}}} \pm {{\textbf {0.02}}}$
INbreast ($n = 82$)	CC	Otsu	$58.16 \pm 0.13$	$45.31 \pm 0.05$
		FCN	$65.93 \pm 0.14$	58.77± 0.12
		U-net	$70.87 \pm 0.08$	$62.85 \pm 0.07$
		MTLSegNet	${{\textbf {72.13}}} \pm {{\textbf {0.09}}}$	${{\textbf {63.99}}} \pm {{\textbf {0.08}}}$
	MLO	Otsu	$55.36 \pm 0.12$	$40.5 \pm 0.12$
		FCN	$67.23 \pm 0.14$	$57.65 \pm 0.13$
		U-net	$67.50 \pm 0.13$	$57.79 \pm 0.12$
		MTLSegNet	${{\textbf {68.41}}} \pm {{\textbf {0.09}}}$	${{\textbf {58.88}}} \pm {{\textbf {0.08}}}$
	CC-MLO	Otsu	$59.32 \pm 0.05$	$48.16 \pm 0.11$
		FCN	$73.69 \pm 0.10$	$64.39 \pm 0.09$
		U-net	$75.17 \pm 0.08$	$65.97 \pm 0.07$
		MTLSegNet	${{\textbf {75.54}}} \pm {{\textbf {0.07}}}$	${{\textbf {66.55}}} \pm {{\textbf {0.06}}}$
Combined evaluation set ($n = 9582$)	CC	Otsu	$71.56 \pm 0.01$	$68.32 \pm 0.02$
		FCN	$81.60 \pm 0.14$	$74.38 \pm 0.14$
		U-net	$83.32 \pm 0.12$	$75.71 \pm 0.13$
		MTLSegNet	${{\textbf {85.92}}} \pm {{\textbf {0.10}}}$	${{\textbf {78.35}}} \pm {{\textbf {0.11}}}$
	MLO	Otsu	$67.56 \pm 0..5$	$54.16 \pm 0.32$
		FCN	$89.87 \pm 0.02$	$82.35 \pm 0.02$
		U-net	$90.49 \pm 0.02$	$83.32 \pm 0.03$
		MTLSegNet	${{\textbf {91.14}}} \pm {{\textbf {0.02}}}$	${{\textbf {84.28}}} \pm {{\textbf {0.03}}}$
	CC-MLO	Otsu	$74.31 \pm 0.01$	$70.30 \pm 0.12$
		FCN	$89.36 \pm 0.91$	$82.47 \pm 0.22$
		U-net	$90.13 \pm 0.11$	$84.29 \pm 0.17$
		MTLSegNet	${{\textbf {92.20}}} \pm {{\textbf {0.01}}}$	${{\textbf {85.99}}} \pm {{\textbf {0.02}}}$

Best values are in [bold].

**Figure 6:**
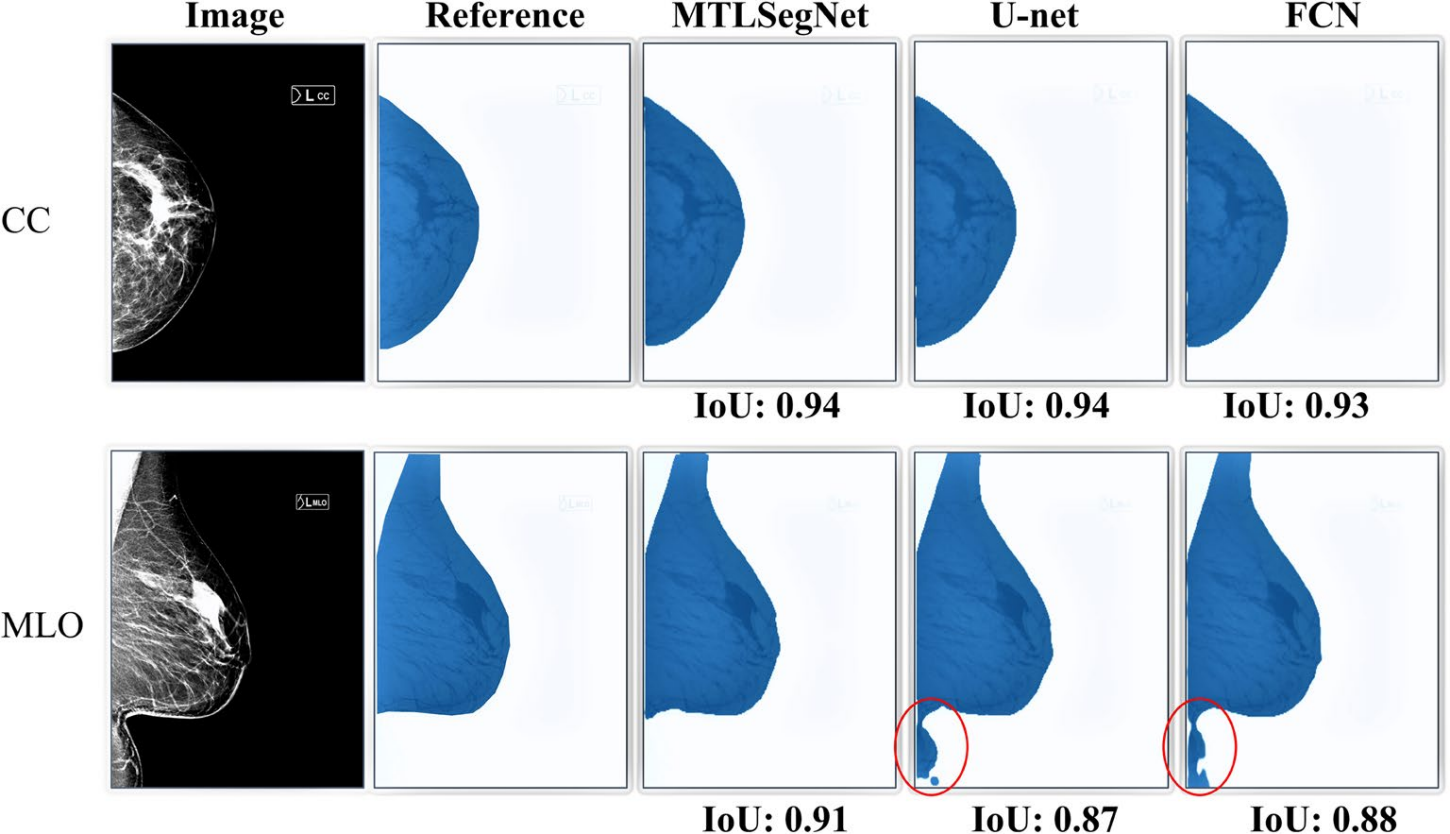
Breast-area segmentation outputs predicted by the MTLSegNet, U-net, and FCN networks on the KUH evaluation data. Red contours point to segmented regions of other tissue (in this case, abdominal tissues) predicted by the U-net and FCN models in the MLO-view.

**Figure 7:**
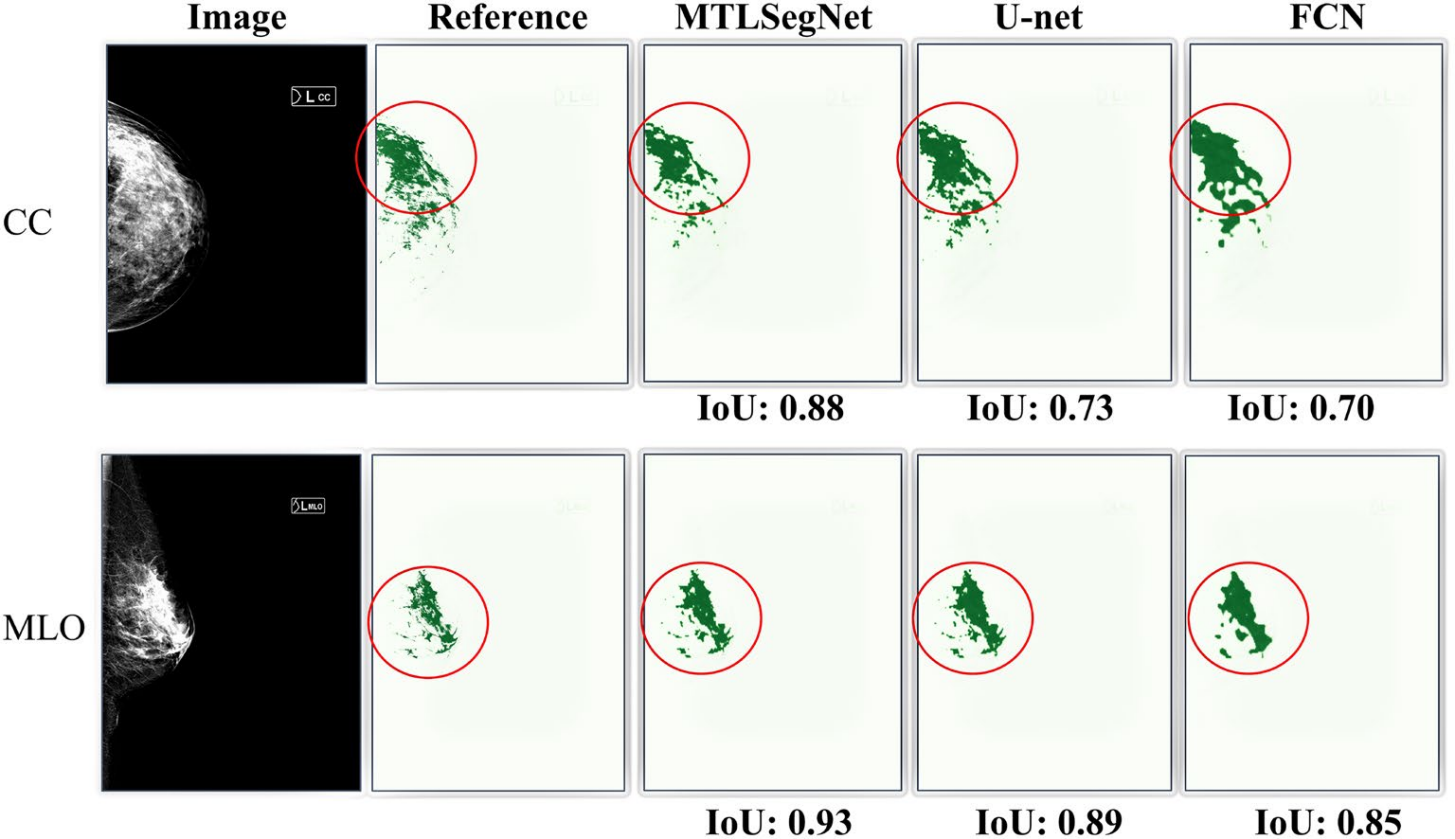
Dense-tissue segmentation outputs predicted by the MTLSegNet, U-net, and FCN networks on the KUH evaluation data. MTLSegNet successfully segmented the dense tissues, while FCN and U-net over-segmented the dense tissues by including the fat tissues within the breast area. For the visualization purposes, we outline the dense-tissue pixel area using the red contour.

Figures 6 and 7 show a few segmentation outputs predicted by the MTLSegNet, U-net, and FCN networks on the KUH evaluation data. For the CC-view mammograms, the breast-area segmentation accuracy for all models is similar (Fig. 6 , first row). For the MLO-view mammograms, MTLSegNet successfully delineated the breast area from other tissues, such as abdominal tissues, as shown in red contours in the second row of Fig. 6.

Figure 7 shows that, for both CC- and MLO-view images, the MTLSegNet model precisely segments the dense tissues by ignoring the fat tissues within the breast area, while the U-net and FCN models included the fat tissues, resulting in over-segmentation. The introduction of dilated convolutions as a bottleneck in the MTLSegNet architecture has potentially improved the dense-tissue segmentation compared to FCN and U-net.

More segmentation examples for qualitative assessment of the MTLSegNet approach are provided in Appendix D.

### Area-based breast percentage density estimation:

#### MTLSegNet more accurately estimates the breast density values compared to the baseline DL approaches

The descriptive statistical summary of the estimated PD values by the baseline models is shown in Table 5. The mean difference between the FCN, and U-net PD values and the radiologist-provided PD values were 0.19%, and 1.9%, respectively, in the CC-MLO-view mammograms. For the KUH evaluation dataset, the average mean difference between MTLSegNet estimated PD values and the reference PD values are similar with a Pearson correlation of $r = 0.90$ (*p* value $< 0.001$). In the CC-view mammograms of KUH evaluation dataset, the FCN estimated PD values are close to the radiologist assessment. The U-net model overestimated the PD values for both CC- and CC-MLO-view mammograms compared with the radiologist assessment. The distribution of density values for the KUH evaluation set are left skewed. Appendix E shows the distribution of the estimated density values on the KUH evaluation dataset for all models.

**Table 5:** Descriptive summary of estimated PD values by the FCN, U-net, and MTLSegNet approaches on the KUH evaluation dataset. We use the mean and the standard deviation (SD) to illustrate the distribution of the PD values and compare the robustness of the model predictions. The number in the brackets denotes the number of mammograms in each view of the KUH evaluation dataset. The last column represents the mean difference between the estimated PD values and the radiologist-provided PD values. We highlighted the model, whose mean difference is close to the reference (radiologist) mean PD value. Lower the mean difference, better the model performance. The negative mean difference indicates that the model has underestimated the PD values compared to the radiologist-provided PD values.

KUH evaluation set ($n = 6840$)			
View	PD estimation	Mean ± SD	$\text {PD}_{\text {model}} - \text {PD}_{\text {radiologist}}$
**CC** ($n = {{\textbf {3,528}}}$)	Radiologist	$10.38 \pm 5.77$	–
	FCN	${{\textbf {10.45}}} \pm {{\textbf {4.85}}}$	0.07
	U-net	$14.17 \pm 6.47$	3.79
	MTLSegNet	$11.28 \pm 5.75$	0.9
**MLO** ($n = {{\textbf {3,312}}}$)	Radiologist	$8.29 \pm 4.62$	–
	FCN	$7.43 \pm 3.71$	$-0.86$
	U-net	$7.94 \pm 3.50$	$-0.35$
	MTLSegNet	${{\textbf {8.61}}} \pm {{\textbf {4.27}}}$	0.32
**CC-MLO** ($n = {{\textbf {6,840}}}$)	Radiologist	$9.42 \pm 5.38$	–
	FCN	$9.61 \pm 4.68$	0.19
	U-net	$11.32 \pm 6.16$	1.9
	MTLSegNet	${{\textbf {9.42}}} \pm {{\textbf {5.28}}}$	0

Best values are in [bold].

Table 6 shows the Pearson correlation coefficient between the radiologist-provided PD and estimated PD from the FCN, U-net, and MTLSegNet models. MTLSegNet shows a higher correlation of $r = 0.90$ [95% CI 0.89, 0.91] (*p* value $< 0.001$) with the radiologist-provided PD values than the FCN and U-net models, with $r = 0.88$ (*p* value $< 0.001$) and $r = 0.84$ (*p* value $< 0.001$), respectively, for the CC-MLO-view mammograms. Additionally, we performed a statistical analysis within the DL approaches. For the CC-MLO-view of the KUH evaluation data, MTLSegNet-estimated PD values are significantly better than FCN and U-net with *p* values $< 0.001$. However, in the CC-view mammograms, FCN- and MTLSegNet-estimated PD values did not show significant difference (*p* value $> 0.01$).

**Table 6:** The Pearson correlation between the estimated PD values and radiologist-provided PD values at 95% confidence interval. For all three CC-, MLO-, and CC-MLO-view mammograms, MTLSegNet shows a higher correlation than the baseline models. Before correlation analysis, we applied a log transform to all density values. We highlighted the model with strongest correlation with the radiologist PD values.

KUH evaluation dataset ($n = 6840$)			
View	PD estimation	*r* *[95% CI]*	*p*-*value*
**CC** ($n = {{\textbf {3,528}}}$)	FCN	0.88 [0.88, 0.89]	$< 0.001$
	U-net	0.87 [0.87, 0.88]	$< 0.001$
	MTLSegNet	**0.90 [0.90, 0.91]**	$< {{\textbf {0.001}}}$
**MLO** ($n = {{\textbf {3,312}}}$)	FCN	0.89 [0.89, 0.91]	$< 0.001$
	U-net	0.86 [0.86, 0.88]	$< 0.001$
	MTLSegNet	**0.91 [0.91, 0.92]**	$< {{\textbf {0.001}}}$
**CC-MLO** ($n = {{\textbf {6,840}}}$)	FCN	0.88 [0.89, 0.89]	$< 0.001$
	U-net	0.84 [0.83, 0.85]	$< 0.001$
	MTLSegNet	**0.90 [0.89, 0.91]**	$< {{\textbf {0.001}}}$

Best values are in [bold].

#### LIBRA and quantra overestimate the breast-density values

Table 7 provides summary statistics of the estimated PD values by the LIBRA, Quantra, and MTLSegNet approaches on the KUH evaluation set for the CC-, MLO-, and CC-MLO-view mammograms. For the CC-MLO-view, the mean PD values estimated by Quantra, LIBRA, and MTLSegNet were $16.18 \pm 15.66$, $14.33 \pm 11.85$, and $9.42 \pm 5.28$, respectively. For the CC-MLO-view mammograms, the mean PD differences between Quantra and LIBRA and the radiologist assessment were 6.76% and 4.91%, respectively, indicating that both Quantra and LIBRA overestimated the PD values (the mean PD value assessed by the radiologists was 9.42% for the CC-MLO-view images). Appendix F shows details of the distribution of the estimated density values on the KUH evaluation set for MTLSegNet, LIBRA, and Quantra. The maximum PD values estimated by LIBRA and Quantra were 62% and 86%, respectively, while the maximum reference PD value in the KUH evaluation set was 48%. In Appendix G, we show a qualitative visualization of the limitations of LIBRA segmentation and compared it with MTLSegNet to illustrate the success of our proposed approach in segmenting the breast area and the dense tissues for more accurate PD value estimation.

**Table 7:** Descriptive summary of estimated PD values of MTLSegNet, LIBRA, and Quantra and the radiologist-assessed PD values using the KUH evaluation set. We show the mean and standard deviation (SD) of the PD values for all models. We highlighted the model, whose mean difference is close to the reference (radiologist) mean PD value. Lower the mean difference, better the model performance.

KUH evaluation set ($n = 6840$)			
View	PD estimation	Mean ± SD	$\text {PD}_{\text {model}} - \text {PD}_{\text {radiologist}}$
**CC** ($n = {{\textbf {3,528}}}$)	Radiologist	$10.38 \pm 5.77$	-
	MTLSegNet	${{\textbf {11.28}}} \pm {{\textbf {5.75}}}$	0.9
	LIBRA	$14.89 \pm 12.34$	4.51
	Quantra	$16.22 \pm 15.53$	5.84
**MLO** ($n = {{\textbf {3,312}}}$)	Radiologist	$8.29 \pm 4.62$	-
	MTLSegNet	${{\textbf {8.61}}} \pm {{\textbf {4.27}}}$	0.32
	LIBRA	$13.66 \pm 11.21$	5.37
	Quantra	$16.13 \pm 15.82$	7.84
**CC-MLO** ($n = {{\textbf {6,840}}}$)	Radiologist	$9.42 \pm 5.38$	-
	MTLSegNet	${{\textbf {9.42}}} \pm {{\textbf {5.28}}}$	0
	LIBRA	$14.33 \pm 11.85$	4.91
	Quantra	$16.18 \pm 15.66$	6.76

Best values are in [bold].

Table 8 provides the correlation of estimated PD values from the LIBRA, Quantra, and MTLSegNet approaches with the radiologist PD values. The PD estimated by MTLSegNet strongly correlates with the radiologist-provided PD values. For the CC-MLO-view images, the proposed MTLSegNet model shows a strong correlation of $\textit{r} = 0.90$ [95% CI 0.89, 0.91] with *p* value $< 0.001$, while LIBRA and Quantra show high correlations of $\textit{r } = 0.67$ [95% CI 0.66, 0.68] and $\textit{r } = 0.64$ [95% CI 0.63, 0.65], respectively, with *p* values $< 0.001$. In Appendices F and G, we qualitatively demonstrate the breast and dense-tissue segmentations from LIBRA and compared them with the MTLSegNet approach. LIBRA failed to exclude the pectoral and abdominal tissues from the breast-area segmentation and over-segmented the dense tissues, resulting in an overestimate of the PD values. The Quantra software tool provides only the PD values; thus, we were not able to compare the intermediate visualizations of breast and dense-tissue segmentations. We report the correlation between LIBRA and the radiologist on an evaluation set of 6840 mammograms. The correlation results are in agreement with Lee and Nishikawa^14^, who showed a Pearson correlation of $r = 0.69$ for the CC-MLO-view mammograms on an evaluation set of 91 mammograms. These results indicate that our proposed model more accurately estimates the breast density values than the existing LIBRA and Quantra tools.

**Table 8:** The Pearson correlation computed between the radiologist-provided PD values and the estimated PD values from MTLSegNet, LIBRA, and Quantra at 95% CIs on the log-transformed PD values. The models with high correlation are highlighted. For both CC- and MLO-view mammograms, our proposed approach demonstrated a strong correlation with the radiologist-provided density values.

KUH evaluation set ($n = 6840$)			
View	PD estimation	*r* *[95% CI]*	*p*-*value*
CC ($n = 3528$)	Radiologist Vs MTLSegNet	**0.90 [0.90, 0.91]**	$< 0.001$
	Radiologist Vs LIBRA	0.66 [0.65, 0.68]	$< 0.001$
	Radiologist Vs Quantra	0.64 [0.63, 0.67]	$< 0.001$
MLO ($n = 3312$)	Radiologist Vs MTLSegNet	**0.91 [0.91, 0.92]**	$< 0.001$
	Radiologist Vs LIBRA	0.68 [0.66, 0.67]	$< 0.001$
	Radiologist Vs Quantra	0.64 [0.62, 0.67]	$< 0.001$
CC-MLO ($n = 6840$)	Radiologist Vs MTLSegNet	**0.90 [0.89, 0.91]**	$< 0.001$
	Radiologist Vs LIBRA	0.67 [0.66, 0.68]	$< 0.001$
	Radiologist Vs Quantra	0.64 [0.63, 0.65]	$< 0.001$

Best values are in [bold].

**Figure 8:**
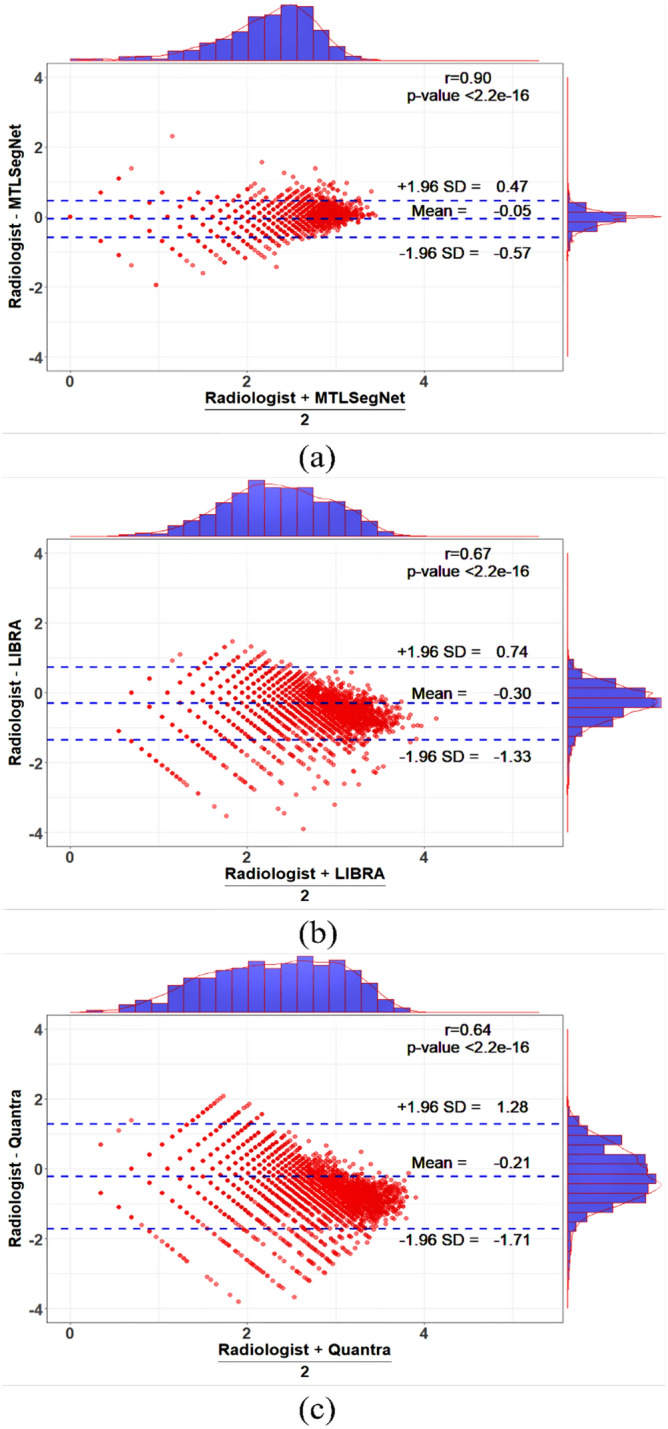
Bland–Altman agreement plots for MTLSegNet, LIBRA, and Quantra with the radiologist-provided PD values for the KUH evaluation dataset. The center blue dotted line shows the average PD values for the two methods. The dotted lines on the top and bottom indicate the LoA range, that is, the value at 1.96 times the standard deviation (SD) in both directions. If the data points are within the LoA, the two methods have strong agreement, otherwise the methods disagree. The Bland-Altman plots are shown for (**a**) MTLSegNet, (**b**) LIBRA, and (**c**) Quantra on the KUH evaluation dataset for the CC-MLO-view mammograms.

Additionally, Fig. 8 shows Bland–Altman agreement plots for MTLSegNet, LIBRA, and Quantra with the radiologist-provided PD values for the KUH evaluation dataset. The estimated PD values using the MTLSegNet approach show a strong agreement with the radiologist (98.6% CDI acceptance range) with LoA from $-0.54$ to 0.52 with a mean bias of $-0.008$ on the log-transformed scale. LIBRA and Quantra show moderate agreement with the radiologist with 82.75% and 80.87% CDI acceptance rates, respectively, and mean biases of $-0.26$ (LoA: $-1.33$ to 0.74) and $-0.21$ (LoA: $-1.70$ to 1.28).

## Discussion

In this study, we developed a DL approach for estimating area-based breast PD value in mammograms using a weight-adaptive multitask learning approach based on 21,315 mammograms from KUH and 10,416 mammograms from the open-source datasets. The results showed that the proposed approach successfully segmented the breast area and the dense tissues and could estimate the breast density with higher precision than the existing LIBRA and Quantra tools. The main reasons for the outstanding performance of MTLSegNet are as follows. First, the model consisted of two task-specific decoders, with the dense-tissue segmentation, as the primary task, and the breast-area segmentation, as the auxiliary task. This architecture helped the model to exclude tissues or organs that adversely affected the segmentation and thus, estimation of the PD values; Second, the proposed model was trained end-to-end with a modified weight-adaptive multitask learning loss function, which enabled the network to generate more accurate predictions; and third, the model was trained using combination of all the training mammograms from all datasets. Datasets had different data distributions; therefore, the model learned from multi-vendor, multi-resolution, and multi-intensity variations, and preferably only a single model was generated for evaluation. Results in Table 4 showed that our model achieved excellent segmentation performance not only for each individual dataset, but also the combined evaluation set of all datasets. The proposed approach successfully segmented the breast area and the dense tissues more precisely than the multitask U-net and FCN approaches by on average, 3.17% and 2.29% relative improvements, in terms of F-score, respectively, in the combined CC-MLO-view data. The estimated PD values using our approach also showed a strong correlation with the values provided by the expert radiologists with a Pearson correlation $r = 0.90$ (*p* value $< 0.001$).

In our study, 6840 out of 7500 mammograms from the KUH evaluation set were within the CDI range and therefore, considered for evaluation. The assessed density values of the excluded 660 mammograms were not in agreement between the radiologists mainly due to the poor quality of the mammograms and blood vessels embedded within the dense tissues. We additionally correlated the PD values estimated by our model with values given by each radiologist for these 660 excluded mammograms. As expected, we obtained moderate correlations with $r = 0.715$ [95% CI 0.706, 0.721] and $r = 0.736$ [95% CI 0.725, 0.746] for such mammograms.

The training sizes of MIAS ($n = 194$) and INbreast ($n = 246$) datasets were considerably lower than KUH ($n = 11{,}052$) and mini-DDSM ($n = 5812$). Additionally, the resolution of mammograms and distribution of pixel intensities were different among the datasets (see Fig. 2). Therefore, the model, trained on the combined training sets of all datasets, has been biased towards datasets having larger training data, i.e., KUH and mini-DDSM. This partly explains that why F-score and IoU values for the MIAS and INbreast datasets were relatively lower compared to the KUH and mini-DDSM datasets in Table 4.

The density assessment in the BI-RADS classification subjects to the radiologist’s experience and often shows intra- and inter-reader variability. Our data-driven DL approach for the density estimation is reproducible, scalable, and furthermore, provides density scores in a continuous percentage scale, which reduces the subjectivity. MTLSegNet accepts mammograms in DICOM format (or any other imaging format) irrespective of the acquisition manufacturer and device model, thus easily scalable. The inference time takes about a minute to estimate density values for 100 mammograms. Our method is, however, able to classify mammograms into BI-RADS density categories. We additionally performed a BI-RADS classification on the INbreast and mini-DDSM evaluation data, where BI-RADS density categories were available. The estimated PD values for these two datasets were categorized into 25% density intervals in line with the BI-RADS 4th edition categories, i.e., 0–25% (category 1), 26–50% (category 2), 51–75% (category 3), and 76–100% (category 4). Our proposed approach successfully classified the INbreast and mini-DDSM evaluation data into BI-RADS categories with accuracies of 87% and 92%, respectively, comparable to the results reported in^56,57^.

Although our proposed approach improves the density estimation, it has a few limitations. The proposed model is restricted to the area-based density estimation. Moreover, due to the stochastic and non-linear nature of the DL methods, it is important to investigate the model uncertainty in predictions, which we leave it to a future study (Supplementary Information).

**Figure 9:**
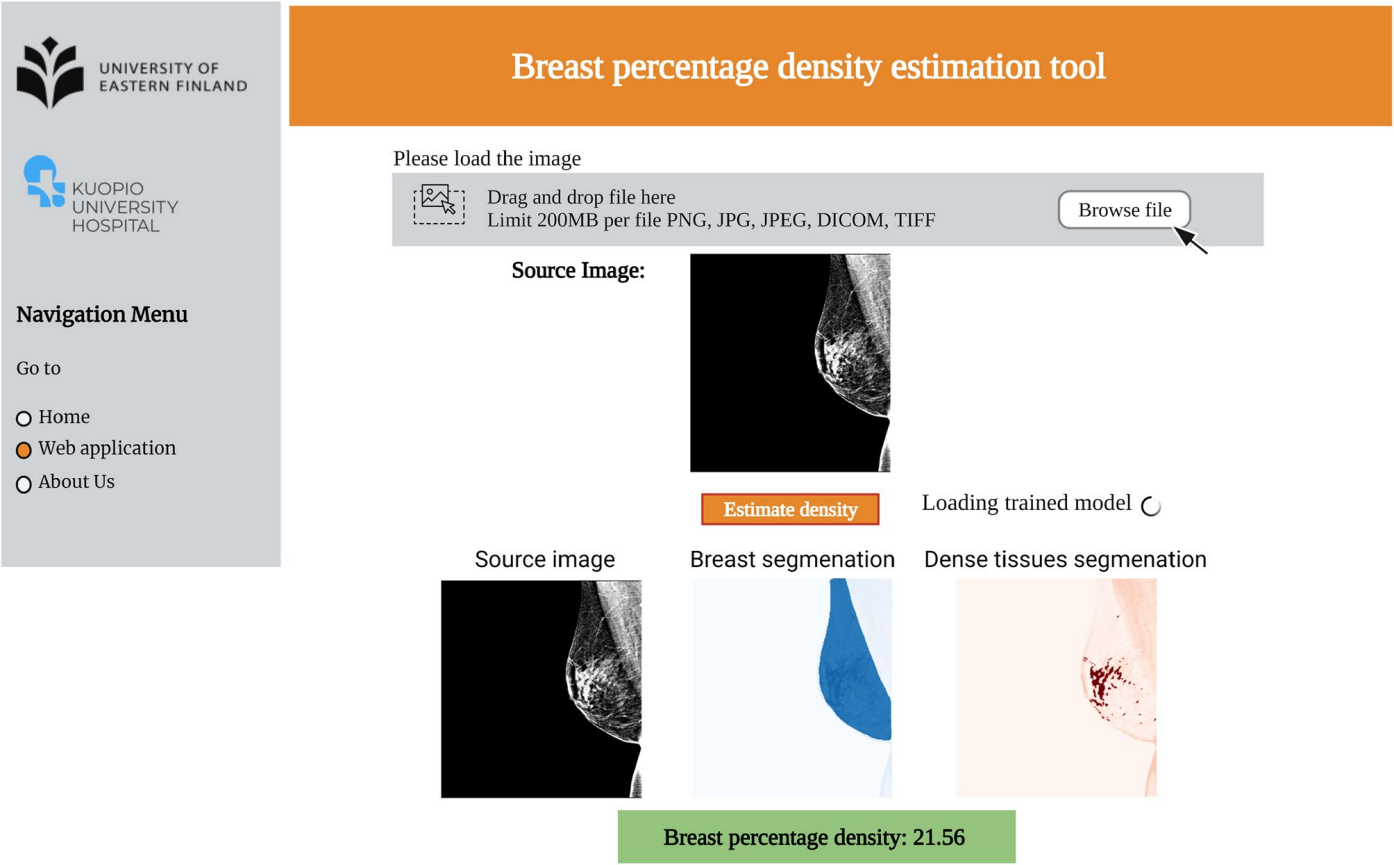
Sample website report of the MTLSegNet PD estimation model.

## Conclusion

To conclude, we developed a reliable and scalable model to estimate area-based breast density from mammograms. The proposed approach showed consistent results and would assist the radiologist in personalized screening settings. In future study, we extend this model to volumetric breast-density estimation by incorporating the concepts of generative adversarial networks to generate the ground-truth segmentations more effectively. This will reduce the time consumed by manually creating the ground-truth segmentations. We will also investigate various uncertainty qualification techniques to improve the model performance and to build trust among radiologist to incorporate the DL algorithms in regular screening workflow. The estimated PD values will be incorporated into BC risk-prediction models in our future studies.

## Supplementary Information

Supplementary Information.

## Data Availability

The KUH imaging data and the corresponding annotations used in this study are available under request to the corresponding author. MIAS and mini-DDSM datasets are open source and publicly available. Annotations of these two datasets are provided in the manuscript GitLab page at https://gitlab.com/rajgudhe.uef/mtlsegnet. INbreast dataset is available with the data owner, contact inesdomingues@gmail.com for more details.

## Code availability

All the implementation source codes, the annotation tool, and the trained models are open-source and freely available online in the manuscript GitLab page at https://gitlab.com/rajgudhe.uef/mtlsegnet. A representative website demo is also shown in Fig. 9. During mammogram upload, the data provider gives consent that the image is used for segmentation and density estimation. We expect this web platform will act as a hub for radiologists to efficiently assess the breast density so that inter- and intra-reader variability are considerably decreased.
