# Supplementary material for: Area-based breast percentage density estimation in mammograms using weight-adaptive multitask learning
Source: Sci Rep. 2022 Jul 14;12:12060. doi: 10.1038/s41598-022-16141-2 (PMC9283472; doi:10.1038/s41598-022-16141-2)
Supplement: Supplementary file 1 — Supplementary Information. [file 41598_2022_16141_MOESM1_ESM.pdf]

Supplementary information

A *Bland-Altman agreement plot between the two radiologists' provided PD values*

Two expert radiologist from KUH have provided PD values for the KUH evaluation set. The two radiologists' PD assessment reliability was computed by inter-reader correlation coefficient at 95% confidence interval. Figure S1 illustrates the agreement using Bland-Altman plot.

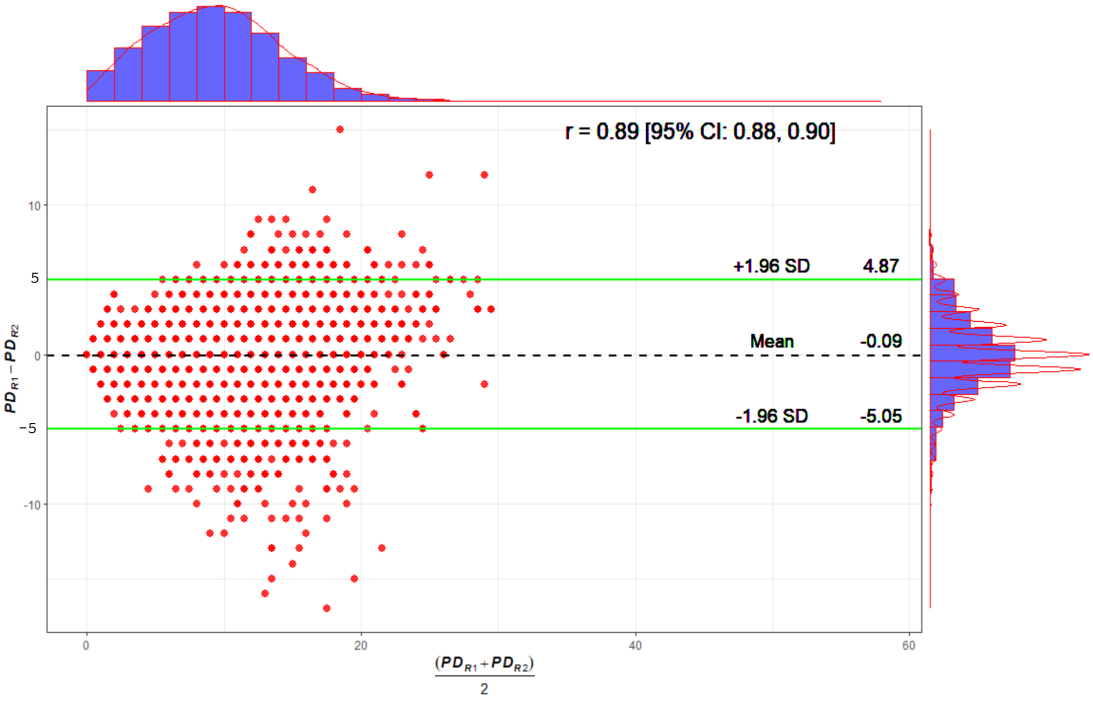

Figure S1. The Bland-Altman plot shows the inter-reader variability between the PD values given by the two radiologists involved in this study. 6,840 out of 7,500 KUH evaluation mammograms are within the CDI range.

B *Finding optimal hyperparameters using the Bayesian optimization search algorithm*

Table S1 presents different hyperparameters included in the Bayesian optimization search algorithm and their found optimal values. In our experiments, we used the multitask segmentation model trained with the Adam optimizer at an initial learning rate of le-3, the "reduced learning rate on plateau" as the learning rate scheduler, and the focal Tversky as the loss function to achieve superior segmentation performance in terms of IoU on the validation set of all the datasets.

Table S1. Hyperparameter tuning using the Bayesian optimization search algorithm. The proposed multitask segmentation is fine-tuned using the validation set of all the datasets. The optimal hyperparameters are highlighted in the last column.

Hyperparameters	Search hyperparameters	Optimal values
Training optimizers	(Stochastic gradient descent[1] , Adam[2] , RMSprop[3])	**Adam**
Learning rate schedulers	(StepLR, MultiStepLR, CosineAnnealingLR, ReduceLROnPlateau, CyclicLR)[4]	**ReduceLROnPlateau**
Initial learning rate	(le-1, le-2, le-3, le-4, le-5)	**le-3**
Loss functions	(BCEwithlogits[5] , Dice[6] , Tversky[7] , focal Tversky[8])	**focal Tversky**

C *Effect of batch size and normalization techniques on the multitask segmentation accuracy*

Figure S2 demonstrates the effect of various normalization techniques at different batch sizes on segmentation performance. We noticed that the normalization techniques degrade the segmentation accuracy with a small batch size of 1 or 2 and further generate the singularity caused by the nonlinear activation function (ReLU) in the convolutional layers of the segmentation models. The multitask segmentation model trained at a batch size of 4 with the combined weight standard and instance normalization (IN) techniques shows superior performance compared with different combinations of batch sizes and normalization techniques. With batch size increased to greater than 4, the combination of weight standard with IN shows consistent performance, while the performances of other normalization techniques degrade slightly. With the larger batch size of 16, the model freezes due to computational memory error (GPU memory). The weight standard technique combined with batch normalization (BN) improved the segmentation accuracy by average relative improvements of 6.17% and 5.88% in terms of IoU and F-score, respectively, compared to the model trained with only BN.

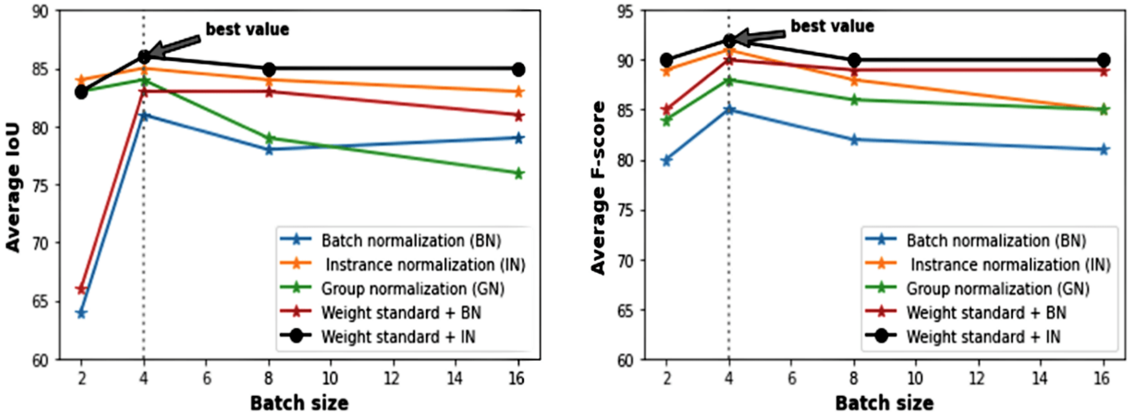

Figure S2. The effect of batch size and weight normalization techniques on the multitask segmentation model (MTLSegNet). The arrow mark in the figure points to the best average value of IoU and F-score, and the dotted line denotes the corresponding optimal batch size.

We trained and evaluated the proposed and the baseline multitask segmentation models with the optimal hyperparameters with a batch size of 4 and the combined weight standard and IN normalization techniques.

D Visualizations of the breast area and the dense tissue segmentations predicted by the MTLSegNet

Here, we show a few examples from all datasets for the breast-area and dense-tissue segmentations predicted by MTLSegNet. We resized all the images to 256x256, and no further pre-processing techniques were applied. The qualitative visualizations are illustrated in Figure S3 and Figure S4 for the CC- and MLO-view mammograms, respectively, on each individual dataset.

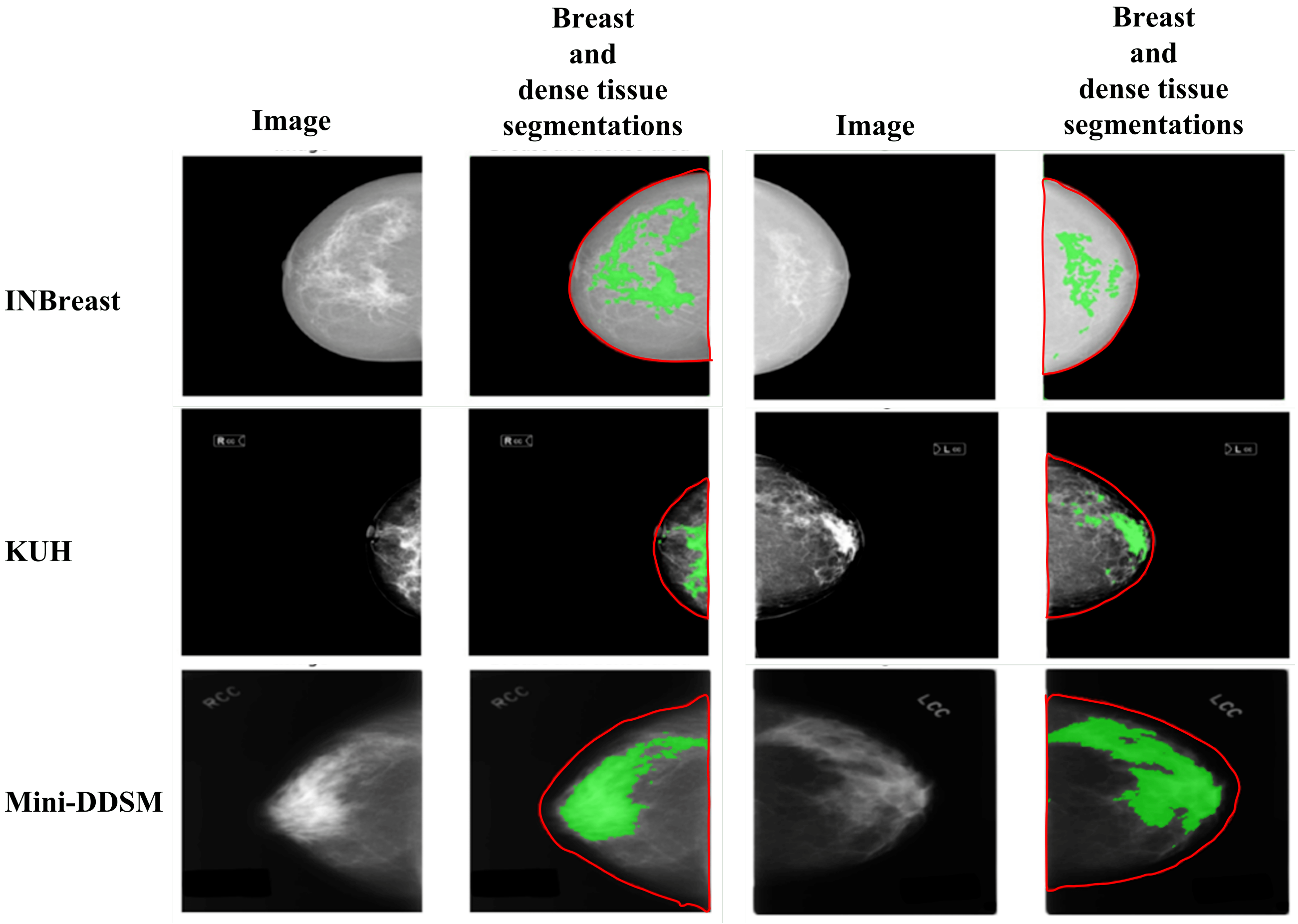

Figure S3. The predicted breast-area and dense-tissue segmentation's on the CC-view mammograms of the evaluation set for the INbreast, KUH, and mini-DDSM datasets. The red contour represents the predicted breast area, and the green pixels represent the predicted dense tissues.

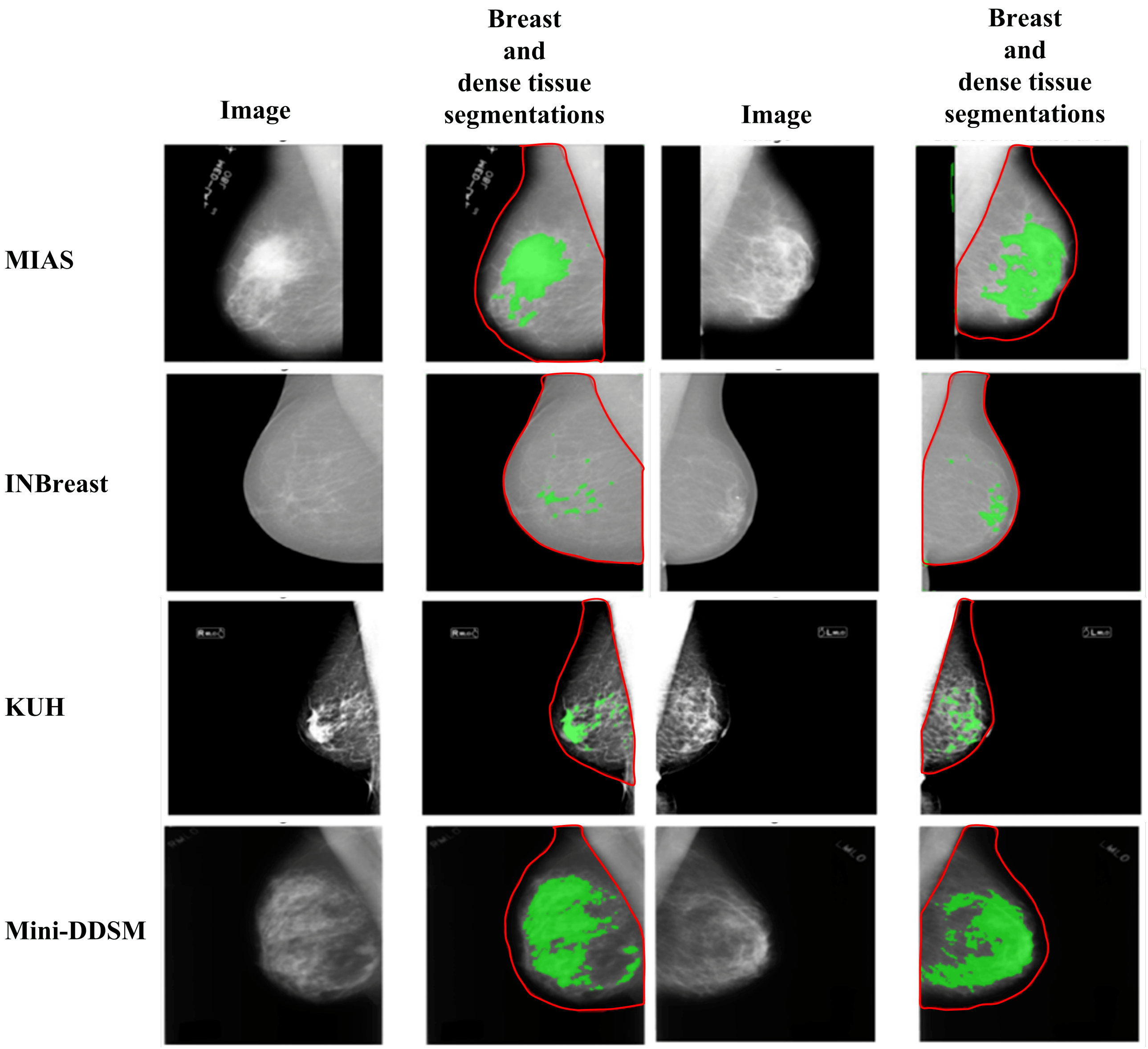

Figure S4. The predicted breast-area and dense-tissue segmentations on the MLO-view mammograms of the evaluation set for the INbreast, KUH, and mini-DDSM datasets. The red contour represents the predicted breast area, and the green pixels represent the predicted dense tissues.

E Distribution of estimated density values on the KUH evaluation dataset using MTLSeg-Net and the baseline DL approaches

Figure S5 shows the density distribution plots of the estimated PD values by the MTLSegNet, FCN, and U-net models on the CC-, MLO-, and CC-MLO-view mammograms of the KUH evaluation dataset. For comparison, the density plots of the radiologist assessments are also given in red.

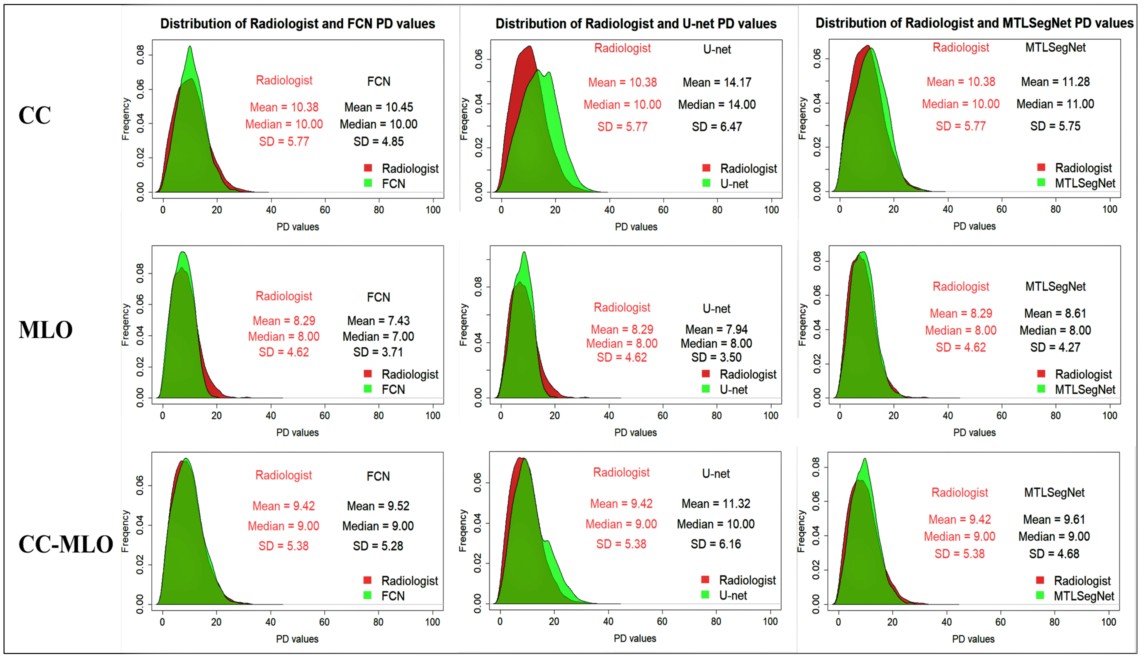

Figure S5. The density distribution plots of the estimated PD values using MTLSegNet, FCN, and U-net on the CC-, MLO-, and CC-MLO-view mammograms for the KUH evaluation dataset. The distribution of the radiologist-provided PD values, as reference, is shown in red

F Distribution of estimated density values on the KUH evaluation dataset using the MTLSegNet, LIBRA and Quantra approaches

Figure S6 shows the density distribution plots of the estimated PD values by the MTLSegNet, LIBRA, and Quantra models on the CC-, MLO-, and CC-MLO-view mammograms for the KUH evaluation dataset. For comparison, the density plots of the radiologist assessments are also given in red.

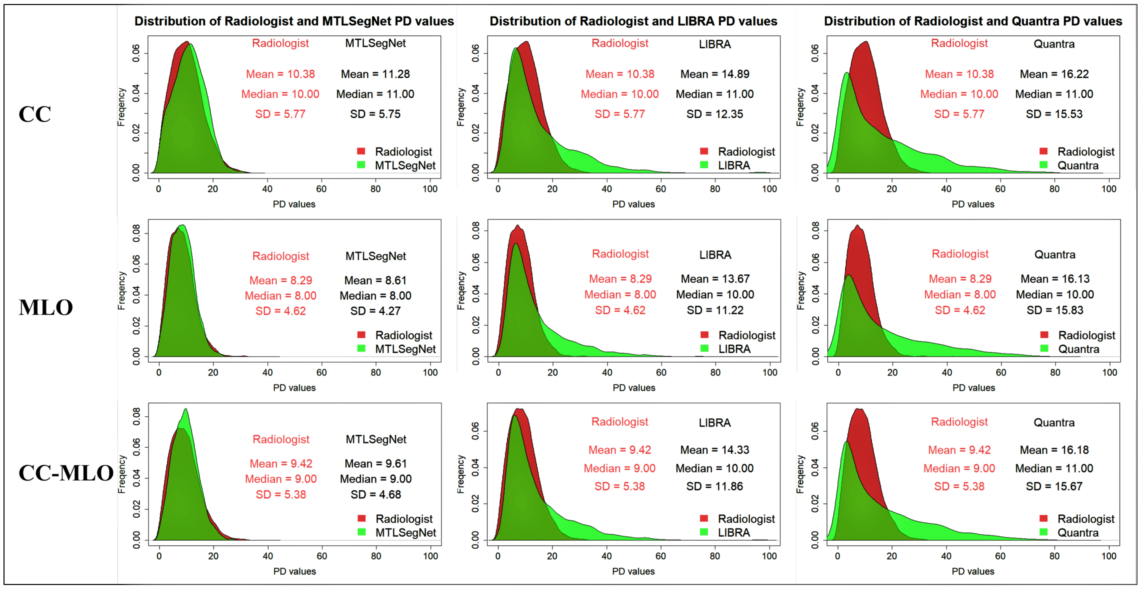

Figure S6. The density distribution plots of the estimated PD values by the MTLSegNet, LIBRA, and Quantra approaches on the CC-, MLO-, and CC-MLO-view mammograms for the KUH evaluation dataset. The distribution of the radiologist-provided PD values, as reference, is shown in red.

G MTLSegNet more accurately segments the breast area and the dense tissues compared to the LIBRA

Figure S7 and Figure S8 visually compare the breast-area and dense-tissue segmentations predicted by the LIBRA and MTLSegNet models for two examples from the KUH evaluation set. We rescaled the output of LIBRA and MTLSegNet to the input mammogram resolution. Figure S7 shows that LIBRA ineffectively segmented the blood vessels as dense tissues, resulting in dense-tissue over-segmentation in the MLO-view mammogram.

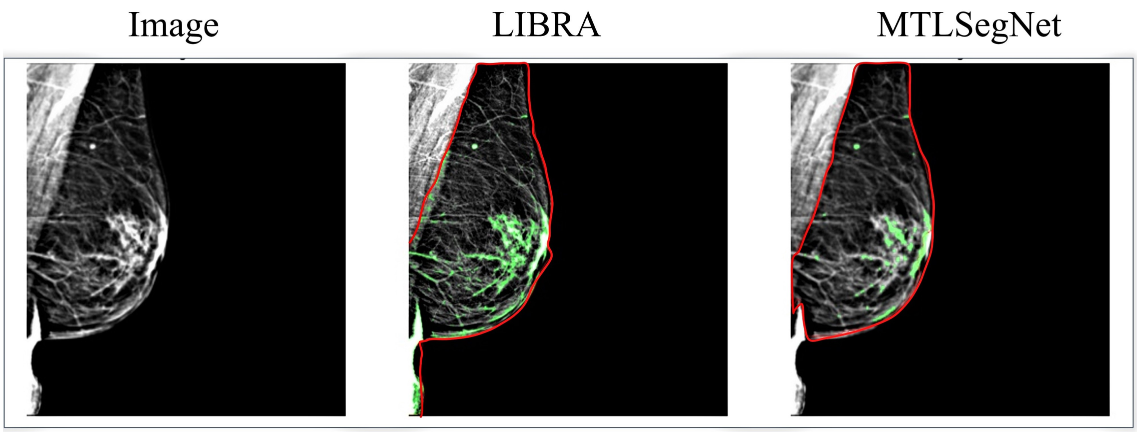

Figure S7. An example showing that LIBRA segmented the blood vessels as fibroglandular tissues, while MTLSegNet successfully discriminated between the blood vessels and the dense tissues within the breast area. Red contours denote the predicted breast area.

The example in Figure S8 shows that MTLSegNet successfully excluded the pectoral muscle and other tissues from the breast-area segmentation. LIBRA often segments the pectoral and abdominal tissues as breast area, thus resulting in an overestimate of breast density by an average of 5% on the KUH evaluation set.

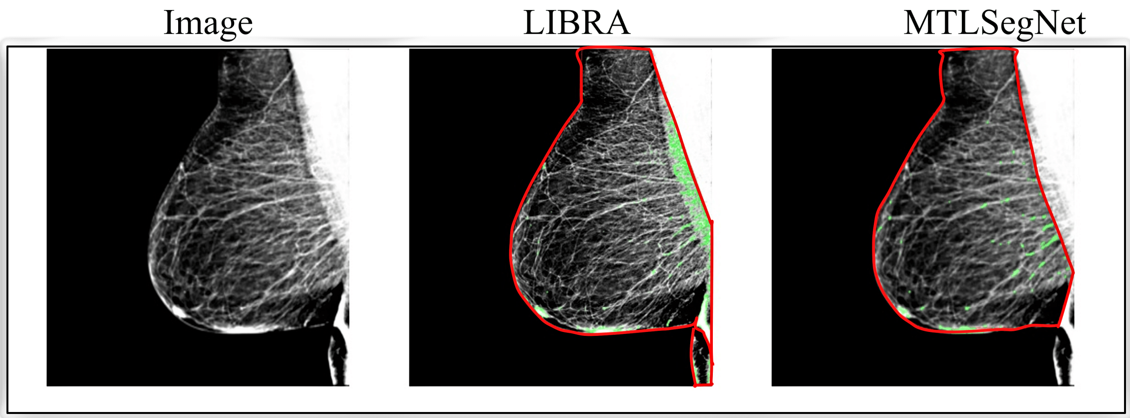

Figure S8. An example demonstrating that LIBRA fails to exclude the pectoral and other tissues from the breast-area segmentation in the MLO-view images. Red contours denote the predicted breast area.

References

1. Amari, S.-i. Backpropagation and stochastic gradient descent method. *Neurocomputing* **5**, 185–196 (1993).
2. Zhang, Z. Improved adam optimizer for deep neural networks. In *International Symposium on Quality of Service* , 1–2 (2018).
3. Ruder, S. An overview of multi-task learning in deep neural networks. *Comput. Res. Repos.* **abs/1706.05098** (2017).

4. How to adjust learning rate. Accessed on 25.02.2022.

5. Buja, A., Stuetzle, W. & Shen, Y. Loss functions for binary class probability estimation and classification: Structure and applications. *Working draft, November 2005* **3** (2003).

6. Sudre, C. H., Li, W., Vercauteren, T., Ourselin, S. & Jorge Cardoso, M. Generalised dice overlap as a deep learning loss function for highly unbalanced segmentations. In *Deep learning in medical image analysis and multimodal learning for clinical decision support*, 240–248 (2017).

7. Hashemi, S. R. *et al.* Tversky as a loss function for highly unbalanced image segmentation using 3d fully convolutional deep networks. *arXiv preprint arXiv:1803.11078* (2018).

8. Abraham, N. & Khan, N. M. A novel focal tversky loss function with improved attention u-net for lesion segmentation. In *International Symposium on Biomedical Imaging*, 683–687 (2019).
